# Artificial Intelligence-Enhanced Remote Monitoring After Cardiac Surgery: A Prospective Single-Centre Feasibility Study with Retrospective Wound Image Classification

**DOI:** 10.3390/jcm15218288

**Published:** 2026-10-24

**Authors:** Jarosław Bis, Kinga Gościńska-Bis, Łukasz Morkisz, Mikołaj Jodłowski, Przemysław Sekuła, Michał Cholewa, Krystian Wita, Marta Strugała, Ryszard Bachowski, Radosław Gocoł

**Affiliations:** 1 Department of Cardiac Surgery, Medical University of Silesia, 40-635 Katowice, Poland; 2 Department of Cardiac Surgery, Upper Silesian Medical Center, Ziołowa 45/47, 40-635 Katowice, Poland; 3 Department of Electrocardiology and Heart Failure, Medical University of Silesia, 40-635 Katowice, Poland; 4 Department of Electrocardiology and Heart Failure, Upper Silesian Medical Center, 40-635 Katowice, Poland; 5 Institute of Theoretical and Applied Informatics, Polish Academy of Sciences, 44-100 Gliwice, Poland; 6 First Department of Cardiology, Medical University of Silesia, 40-635 Katowice, Poland

**Keywords:** remote patient monitoring, cardiac surgery, surgical wound infection, deep learning, explainable artificial intelligence, feasibility study

## Abstract

**Background/Objectives:** Unplanned readmission within 30 days affects 12–20% of patients after cardiac surgery, largely through complications developing at home. We evaluated the feasibility, acceptability, and adherence of a 30-day post-discharge remote monitoring programme. In a separate retrospective analysis that did not inform patient care, we also assessed how accurately an investigational AI-based classifier triages the programme’s wound photographs. **Methods:** In this prospective single-centre study, 76 of 85 screened patients were monitored for 30 days with twice-daily blood pressure and pulse, daily weight, electrocardiography (ECG), and alternate-day wound photographs, all reviewed daily by a study clinician. Retrospectively, 735 photographs of 88 wounds from 71 patients, labelled by two blinded clinicians, were used to train an ensemble of five convolutional neural networks, tested on a patient-disjoint test set of 147 images from 15 patients. **Results:** All 76 eligible patients enrolled and 75 (99%) completed the programme, with 82% adherence to the core measurements and an acceptability score of 6.3 (SD 0.5) of 7. Daily review produced 494 teleconsultations, 10 emergency referrals, and 6 admissions (7.9%), and atrial fibrillation was confirmed in 18 episodes in 11 patients. Agreement between the two wound labelling clinicians was κ 0.89. On the test set, the classifier achieved an area under the precision–recall curve of 0.747 (95% CI 0.402–1.000), image-level sensitivity 73.3% (11/15; 95% CI 33.3–100%), precision 73.3%, and wound-level sensitivity 5/6; the one confirmed wound complication in the test set was flagged. **Conclusions:** Intensive remote monitoring with daily clinician review was feasible after cardiac surgery and was sustained by patients. Daily review identified arrhythmias and wound problems, most of which were managed remotely or in the outpatient clinic. The classifier is a proof of concept for automated triage of such photographs by reproducing clinicians’ visual judgment; given the wide confidence intervals, larger multicentre validation is needed.

## 1. Introduction

Cardiac surgery is currently considered the most complex of all surgical domains [1,2] due to patient comorbidities, procedural difficulty, and the need for optimal multidisciplinary coordination. Success in dealing with these challenges translates into early recovery of patients and better outcomes in terms of in-hospital care.

Excellent in-hospital outcomes can, however, be thwarted by sub-optimal care post-discharge home. During the first weeks after leaving the hospital, patients suffer from various degrees of cardiorespiratory distress, metabolic destabilisation, mobility impairment, and mental “shock” after surgery [3,4,5]. Given these conditions, the effectiveness of standard outpatient care in preventing excess morbidity may be limited. It has been demonstrated that unplanned hospital readmissions within 30 days post-discharge range from 12 to 20% and increase long-term mortality [6,7]. Interestingly, the majority of 30-day readmissions are caused by potentially preventable reasons such as wound site infections, congestive heart failure, arrhythmias, or pleural effusion [8,9].

Several solutions, including post-acute care facilities and home healthcare visits, have been proposed to improve the quality of post-discharge care [10,11]. Their widespread implementation is limited, however, by logistical constraints, insufficient resources, and high costs.

Over the last decade, digital health services have emerged as useful tools in managing the health of patients living at home [12]. The outbreak of the 2020 COVID-19 pandemic has accelerated the development of these services, particularly remote monitoring systems, which enable the measurement and transmission of various health parameters with the use of devices operated by patients at home [13,14]. Remote systems have been recently shown as useful and effective in the domains of cardiology and cardiac surgery for the monitoring of patients after acute coronary syndromes and surgical interventions [15,16]. The benefits of digital health services can be extended further by co-opting artificial intelligence (AI) for analysis and assessment of acquired data, particularly medical images [17,18]. AI has been shown to perform as well as trained human clinicians in pattern recognition tasks, and even outperform them when consistency and processing of large datasets is required [17,19]. More specifically, image-based artificial intelligence has recently been applied to surgical wound photographs on large, predominantly retrospective datasets [20,21].

Given the limitations of standard post-discharge home care in preventing excess morbidity and high readmission rates, in this study, we assessed the feasibility and acceptability of an integrated programme combining patient self-measurements with AI-assisted electrocardiogram (ECG) screening in the 30-day post-discharge period following cardiac surgery, and, separately, we evaluated retrospectively whether an investigational AI-based wound image classifier can triage the surgical wound images that such a programme collects.

This study makes two contributions. Clinically, it shows that intensive, clinician-supervised remote monitoring after cardiac surgery is practicable, that patients stay with it, and that daily review identifies complications that can then be managed, most of them without hospital admission. Technically, it provides a proof of concept that the wound photographs that such a programme collects can be triaged automatically, so that—given enough annotated inflamed cases—clinicians would not need to review every image; validation on a larger, multicentre dataset is the next step, and the programme itself supplies the data for it.

## 2. Materials and Methods

### 2.1. Study Design

This prospective study was performed in a large tertiary cardiac surgical centre in Poland.

As a single-arm prospective feasibility study rather than a clinical trial of a healthcare intervention, the study did not require prospective registration in a trial registry. This study is reported in accordance with the TRIPOD+AI reporting guideline [22].

Patients who underwent cardiac surgery from March 2024 to November 2025 were considered for participation. Eligibility required access to a camera-equipped smartphone, capability of operating the remote monitoring devices, discharge home between 7 and 10 days post-surgery and agreement to participate in teleconsultations. Patients addicted to psychoactive substances were excluded.

### 2.2. Recruitment and Feasibility Outcomes

Patients were approached during the final days of their postoperative stay whenever a monitoring set was available; recruitment was therefore determined by device availability rather than consecutive. Approached patients received a written study summary and had the opportunity to ask questions, and they were asked whether they felt they were able to take and send wound photographs and to operate the monitoring devices. Patients who reported that they did not feel able to do so were considered ineligible. Feasibility was assessed descriptively in five domains, without prespecified success thresholds: (i) eligibility and recruitment—the proportions of screened patients who were eligible and of eligible patients who consented; (ii) retention—the proportion completing the 30-day programme; (iii) adherence—measurements performed as a proportion of those prescribed; (iv) technical performance—device malfunctions and transmission failures; and (v) acceptability—patient feedback on a study-specific questionnaire. Adherence to blood pressure/pulse and weight measurements was calculated over the first 28 days of monitoring (four complete weeks), which allowed week-by-week comparison; the final days of the programme, on which the equipment was returned and the closing outpatient consultation took place, were not included. The denominator was the number of prescribed measurements during patient-days at risk, excluding days of hospitalisation, during which monitoring was suspended, and days from the day of death onward. Adherence to wound photography was calculated against the 15 photographs prescribed per patient over the 30-day programme.

### 2.3. Ethics Approval and Consent to Participate

The study protocol was approved by the Bioethics Committee of the Medical University of Silesia, Katowice, Poland (decision BNW/NWN/0052/KB1/9/I/24, dated 19 March 2024). All participants provided written informed consent before enrolment. The consent covered participation in the monitoring programme and the retrospective use of the collected data, including wound photographs, for the development and evaluation of image analysis algorithms and for publication.

### 2.4. Study Procedure

On discharge, eligible patients provided informed consent and received detailed device training from our telemedicine department staff.

### 2.5. Monitored Health Parameters/Devices

Electrocardiogram (ECG)—One of two ECG-monitoring device types was used. The first was a 6-lead personal handheld ECG device (Kardia Mobile 6L, AliveCor, Mountain View, CA, USA) paired with the Kardia app on the patient’s smartphone. Patients were asked to record a 30 s ECG sequence 3 times a day (morning, noon, evening) and additionally every time they felt potential symptoms of arrhythmia (fast, slow and/or irregular heart beats, dizziness). The preliminary assessment was performed by the Kardia AI algorithm and then sent to the KardiaPro telemedicine platform, where diagnosis was confirmed by medical staff specialised in cardiac arrhythmias monitoring, in a 24/7 telemonitoring centre, which reviewed uploaded recordings around the clock and notified the study staff of detected arrhythmias. The second option was a 3-lead pocket ECG (Medicalgorythmics, Warsaw, Poland) with self-adhesive electrodes and an AI algorithm for preliminary diagnosis, which was verified by the medical staff conducting this study. Patients were asked to wear the system throughout the study period, except for hygienic procedures like washing and bathing. Pocket ECG was the first choice for patients with a higher risk of atrio-ventricular block and for those who did not agree to install the Kardia app on their smartphone.

The ECG algorithms built into both devices are commercially available and have been evaluated in published studies [23,24]; the present study was not designed to assess their diagnostic accuracy. Atrial fibrillation (AF) was defined as an ECG recording showing AF for at least 30 s [25]; the duration of Kardia 6L recordings was set to 30 s for this reason. All Kardia 6L recordings, including those classified as normal by the algorithm, were reviewed by the staff of an external 24/7 telemonitoring centre. Pocket ECG was operated in telemetry mode with the device’s default settings and monitored by the study staff during working hours: events classified as critical by the device algorithm, which for AF meant AF with a fast or slow ventricular rate, were transmitted in real time; AF at a normal ventricular rate and other arrhythmias were identified from the report generated and reviewed daily, which was of the kind produced in standard ambulatory (Holter) ECG monitoring and included all detected arrhythmias and AF burden. A summary report was generated at the end of monitoring. For both devices, clinically relevant findings were referred to a study cardiologist, who confirmed the final diagnosis. AF was counted in episodes: with pocket ECG, a continuous period of AF was one episode; with Kardia 6L, consecutive recordings showing AF were counted as a single episode until a recording showed sinus rhythm.

Arterial blood pressure (ABP) and pulse—An upper-arm iXellence BPM Home IT device (Genexo, Warsaw, Poland) was used. Patients were advised to measure their ABP and pulse 2 times per day (morning and evening).

Body weight—A Charder MS6110 medical floor scale (Charder Medical, Taichung City, Taiwan) was used, and patients were advised to self-weigh daily before breakfast.

International normalised ratio (INR)—Only in patients after implantation of mechanical valvular prostheses on treatment with vitamin K antagonists. The Qlabs Electrometer system (Micropoint, Shenzhen, China) was used. Patients were asked to measure INR on the second day after discharge home. Subsequent measurements were performed by the patient following the instructions of the staff.

Healing of surgical wounds—Patients photographed their surgical wounds with their smartphone every other day.

ABP, pulse, body weight, and INR data were transferred in real time via a Bluetooth hub (Pro-PLUS, Warsaw, Poland) to the telemedicine platform. The photographs were sent by the patient using the Multimedia Messaging Service to a study-dedicated smartphone, which was kept by the study team and protected by password access control; images were transferred to the secured study platform and deleted from the handset. All data associated with the study were handled and stored in accordance with the General Data Protection Regulation.

All health parameters collected in the study were reviewed once a day, during working hours and including weekends, by the study clinicians (four cardiac surgeons and two cardiologists). Following the review, when considered adequate, a teleconsultation with the patient was initiated by the clinician. Teleconsultations were initiated either in response to findings on daily review or, in patients without such findings, as supportive contacts to reinforce adherence to pharmacotherapy and physical activity recommendations. Escalation decisions were made by the reviewing clinician on the basis of clinical judgment, in line with standard postoperative practice; no prespecified numerical thresholds or decision algorithms were used. The teleconsultations covered such health-related issues as: management and adjustment of pharmacotherapy, pain management, warfarin dose adjustment, recommendations on physical activity, fluid balance control, and surgical wound care instructions. Three outcomes of teleconsultations were possible depending on the risk assessment: remote medical advice and support, recommendation for a visit in an outpatient clinic, and recommendation for a visit in a hospital emergency department.

Patients could contact the study team by phone or text message for study-related concerns; inquiries were reviewed daily and, when needed, followed by a clinician-led teleconsultation.

Patients were informed that the participation in this study does not include medical care in health emergencies; in the case of such an event, they were advised to call the emergency ambulance number.

The duration of the remote monitoring was set at 30 days in each patient. On the last day, the patient returned the study equipment and had an outpatient consultation with a cardiologist including transthoracic echocardiography. Acceptability was assessed at the closing visit with a 15-item questionnaire developed by the study team for this study on the basis of the Technology Acceptance Model and the Unified Theory of Acceptance and Use of Technology [26,27]; it has not been formally validated. The questionnaire was administered in Polish, handed out by a study cardiologist, and completed anonymously by the patient without assistance. The English translation (File S1) was verified by a native English speaker. Each item was rated on a 7-point Likert scale (1 = strongly disagree, 7 = strongly agree).

### 2.6. Statistical Analysis

The prospective monitoring data were analysed descriptively, without hypothesis testing. Continuous variables are presented as mean ± standard deviation or median (interquartile range) and categorical variables as number (percentage). The 95% confidence interval for Cohen’s κ was derived from its large-sample standard error. Statistical analyses were performed in R version 4.6.1 (R Foundation for Statistical Computing, Vienna, Austria); the wound image classifier was implemented in Python 3.15.0 with PyTorch 2.14.1 and scikit-learn 1.9.1.

### 2.7. Investigational AI-Based Wound Image Classifier

Two distinct kinds of algorithm are involved in this study. The ECG algorithms built into the two monitoring devices (Kardia AI and the pocket ECG algorithm, described above), which provided AI-assisted ECG screening, are off-the-shelf components of the monitoring solution; they screened recordings as they arrived, every finding was verified by clinical staff, and their output informed patient management. The investigational AI-based wound image classifier described in this section is our own and served a different purpose: it was developed and evaluated retrospectively on the stored photographs, as a proof of concept, and did not contribute to any clinical decision during the monitoring programme.

We performed this exploratory analysis to evaluate whether automated screening could help prioritise cases for clinician attention. A neural network image classifier was trained and assessed for this task. To support clinical interpretability, we also conducted an explainability analysis, checking whether the image regions that drive the model’s output coincide with the wound regions that clinicians consider relevant.

### 2.8. Task and Data

We frame inflammation assessment of surgical wounds as a binary image classification problem with classes Good (index 0) and Inflamed (index 1), the latter denoting a wound judged to show inflammation. The classification target is therefore the clinicians’ binary visual judgment of whether the wound appears inflamed on the photograph. The reference standard external to the image, used only for the confirmed complication analysis (Results), is the record of surgical intervention and microbiological culture: confirmed wound complications were defined by three criteria together, namely surgical debridement in the outpatient clinic, a positive bacterial culture, and antibiotic treatment. Each photograph was graded independently by two specialist cardiac surgeons, each with at least 5 years of post-specialisation experience, blinded to each other’s assessment and to the wound’s clinical outcome, using the Southampton wound assessment scale [28]. Photographs graded IId or higher (erythema with other signs of inflammation around the wound, discharge, pus, or deep infection) were labelled Inflamed and lower grades Good; all labelling preceded model development, so the labels are fixed inputs the classifier could not have influenced. Pre-adjudication agreement between the two readers was quantified by Cohen’s κ, positive specific agreement, and Gwet’s AC1 (values reported in Results). The 16 discordant images (2.2%) were adjudicated by a third specialist cardiac surgeon, whose consensus labels served as the reference standard for training and evaluation. The primary (study) dataset contains 735 images (657 Good, 78 Inflamed) from 71 patients. The images were split at the patient level, so that all photographs of a patient belong to the same partition. A test set of 147 images (20%; 132 Good, 15 Inflamed) from 15 patients is separated first and is never used for training, screening, or hyperparameter optimisation; only the final five-model ensemble is evaluated on it, once all model-selection decisions have been completed. Model development—screening, hyperparameter optimisation, and validation—is then carried out on the remaining 588 images (525 Good, 63 Inflamed) from 56 patients by 5-fold cross-validation over five patient-disjoint folds of 116–122 images (11–17 Inflamed each): each fit trains on four folds and validates on the fifth. The data are strongly class-imbalanced: the positive (Inflamed) class accounts for only 10.2% of the test set and 9.5–13.9% of each fold. These 735 images are a subset of the 749 photographs received during the monitoring programme and cover 88 distinct wounds of 71 patients. All photographs were taken and submitted by the patients themselves; some were taken during outpatient visits, for example to a general practitioner, which accounts for the clinical setting visible in some images. The remaining 14 photographs (1.9%) from 6 patients were excluded: nine from the four patients with confirmed wound complications, because they were taken after surgical debridement, when the treated wound no longer represented the condition the classifier is intended to triage; and 5 photographs from two patients, because their quality was insufficient for reliable assessment (for example, blurring, inadequate framing or lighting, or the wound being obscured by a dressing). No patient or wound was excluded entirely. A total of 28 of the 88 wounds, from 28 patients, provide the Inflamed images, and most inflamed examples are leg (vein-harvest) rather than sternotomy wounds. A wound is defined as one surgical site of one patient, either the sternotomy or the leg (vein-harvest) wound. The 88 wounds comprise 57 sternotomy and 31 leg wounds; a total of 54 patients contributed one wound and 17 contributed both a sternotomy and a leg wound, and the number of photographs per patient had a median of 7 (interquartile range 1–14; range 1–53). Each patient with an inflamed wound contributed exactly one inflamed wound. The test set contains 19 wounds of its 15 patients, 6 of them inflamed, and the development set 69 wounds of its 56 patients, 22 of them inflamed.

To probe whether external wound imagery improves the classifier, we optionally enrich the training part of each fold only with two additional datasets, leaving the validation folds and the test set byte-for-byte identical to the study-only baseline. This guarantees that all reported numbers are directly comparable across configurations. Neither external dataset is graded on the Southampton scale used for the study images, so their annotations had to be mapped onto our two classes. The mapping below agreed with the study clinicians after a preliminary visual review of the external images. The mapped labels are a proxy for our reference standard, not a re-grading against it, which is one reason why the external images are used only for training and never for validation or testing. The additional sources and their label rules are:

- SurgWound [29] (686 images: 573 Good, 113 Inflamed), labelled from the infection_risk annotation (Low → Good; Medium/High → Inflamed).
- RedScar [30] (394 images: 366 Good, 28 Inflamed), labelled from the filename infection flag (infection = 0 → Good; infection = 1 → Inflamed).
- RedScar-Inflamed, a variant that appends only the 28 Inflamed RedScar images, motivated by the observation that RedScar’s 366 out-of-domain Good images dilute the training set.

### 2.9. Backbones and Training

We evaluate two ImageNet-pretrained backbones from the timm library [31]: Xception [32] (input 299 × 299) and EfficientNet-B4 [33] (input 380 × 380). Each backbone is used as a feature extractor followed by a single linear classification head. Training proceeds in two phases. During a warm-up phase the backbone is frozen and only the head is trained; thereafter, the last *N* top-level children of the backbone are unfrozen and fine-tuned with a smaller learning rate, using two parameter groups (head and unfrozen backbone). We optimise with Adam and a class-weighted cross-entropy loss (see below). Training runs for at most 30 epochs with early stopping on the fold’s validation loss (patience 5 epochs, counted from the first fine-tuning epoch). Each run is executed on a single GPU, and all runs are tracked with MLflow. Key hyperparameters are summarised in Table 1.

### 2.10. Data Augmentation

All images (study and additional datasets, in every partition) are first rotated according to their EXIF (exchangeable image file format) orientation tag, resized to the backbone’s input size with the aspect ratio preserved (letterboxing: the image is scaled to fit and centred on a black square canvas), and colour-normalised with Shades-of-Gray white balance (Minkowski norm *p* = 6) [34], computed on the image before padding. Augmentation is applied to the training images of each fold only; validation and test images receive only this preprocessing and normalisation. The training pipeline consists of a random resized crop of the letterboxed image (covering 90–100% of the image area with aspect ratio in [0.9, 1.1]), a random horizontal flip, a random rotation of up to ±10°, a random translation of up to ±5% of the image dimensions, and colour jitter (brightness and contrast each up to 0.2, saturation up to 0.1, no hue change). All images are normalised with ImageNet statistics.

### 2.11. Handling Class Imbalance

Given the ≈10% positive prevalence, we weight the cross-entropy loss by inverse class frequency, *w_c_* = *M*/(*C n_c_*), where *M* is the number of training samples, *C* = 2 the number of classes, and *n_c_* the count of class *c*. On a study-only training fold (420 Good and 46–52 Inflamed images, depending on the fold), this yields weights of 0.55–0.56 (Good) and 4.54–5.07 (Inflamed).

### 2.12. Checkpoint Selection and Evaluation

Because the task is a rare-positive screening problem, we select the saved checkpoint by the validation area under the precision–recall curve (AUPRC, equivalently average precision) of the Inflamed class, rather than by a threshold-dependent quantity such as accuracy or balanced accuracy. AUPRC is threshold-free, focuses on the positive class, and is smoother than hard prediction metrics on a validation fold with only 11–17 positives, which makes checkpoint selection more stable and aligns the selection criterion with the primary evaluation metric.

For model screening and selection, we use patient-disjoint 5-fold cross-validation on the 588-image non-test pool and rank configurations by mean cross-validated validation AUPRC; the 20% test split is held out and used only to evaluate the final tuned model, so screening and hyperparameter optimisation never see the test set. We report AUPRC (primary) and ROC-AUC as threshold-free discrimination metrics, together with operating point metrics at the default decision threshold: balanced accuracy, Inflamed recall (sensitivity), Inflamed precision, Inflamed F_1_, and overall accuracy. The no-skill AUPRC baseline equals the positive prevalence (≈0.10). AUPRC is computed as average precision (scikit-learn average_precision_score), AP = Σ*_n_* (*R_n_* − *R_n_*_−1_) *P_n_*, where *P_n_* and *R_n_* are the precision and recall at the *n*-th decision threshold; unlike the trapezoidal area under the precision–recall curve, it does not interpolate linearly between operating points, which can overestimate the area.

### 2.13. Statistical Analysis of Results on Test Set

To calculate uncertainty in the classifier’s performance, we estimated non-parametric 95% confidence intervals using 5000 stratified patient-level cluster bootstrap iterations. Patients were resampled with replacement within groups separately for patients with and without an inflamed wound, retaining all photographs from each selected patient. Performance metrics were calculated for each iteration using the predictions of the classifier. Confidence limits were obtained from the 2.5th and 97.5th percentiles of the bootstrap distributions. This accounts for correlation between multiple photographs taken of the same patient over time.

### 2.14. Hyperparameter Optimisation with Optuna

Optuna [35] is an open-source hyperparameter optimisation framework. Instead of exhaustively scanning a grid, it casts tuning as sequential model-based (Bayesian) optimisation: its default sampler, the Tree-structured Parzen Estimator (TPE) [36], maintains probabilistic models of the objective for the “good” and “poor” hyperparameter values observed so far and preferentially proposes new configurations in regions likely to improve the objective. This makes it markedly more sample-efficient than grid or random search—important here, because each evaluation is a full *K*-fold cross-validation (i.e., *K* separate model fits) and is therefore costly. Optuna additionally exposes a define-by-run search space and stores every trial in a shared relational database, so many workers can optimise a single study concurrently. We chose it for exactly these reasons: sample efficiency under an expensive objective, effortless multi-GPU parallelisation through a shared PostgreSQL study, and reproducible, inspectable trial histories.

### 2.15. Screening, Selection, and Tuning Procedure

Compute is spent in three deliberate stages:

1. Screening (12 models). We first evaluate the full 6 × 2 grid of training data configurations (study images only; +RedScar; +SurgWound; +RedScar+SurgWound; +RedScar-Inflamed; +RedScar-Inflamed+SurgWound) crossed with the two backbones, using the default hyperparameters of Table 1 and AUPRC checkpoint selection. Each configuration is assessed by patient-disjoint 5-fold cross-validation on the non-test pool (the 20% test split is held out and never used here), giving a robust, leakage-free estimate of validation performance for each backbone/data combination.
2. Selection. Configurations are ranked by mean cross-validated validation AUPRC (reported in Results); the test set plays no role in selection. On this basis, EfficientNet-B4+SurgWound (validation AUPRC 0.652) and EfficientNet-B4+RedScar+SurgWound (0.650) are the two top configurations; because they differ far less than the fold-to-fold standard deviation (0.10–0.11), both are carried forward for tuning. Optimising only the two leading configurations, rather than all twelve, concentrates effort on the models we would actually deploy.
3. Hyperparameter optimisation. We tune each of the two configurations with Optuna in a separate study: 100 trials, TPE sampler, and objective = the mean 5-fold cross-validated validation AUPRC. Cross-validation makes the objective robust to the small positive count, and the project test set is never touched inside the search. The search space is listed in Table 2; all other settings (max epochs, early stopping, class weighting, augmentation, seed) are held fixed. Within the hyperparameters of each study’s best-AUPRC trial, a fresh 5-fold cross-validation is run, which gives the tuned model’s validation performance. Subsequently, one of the two tuned configurations is retained as the final model (see Results). The final model is an ensemble of the five fold-specific models of this cross-validation—each trained on four folds, with its checkpoint selected by validation AUPRC on the fifth—whose predicted Inflamed probabilities are averaged and thresholded at 0.5; no model is retrained on the pooled folds. The final ensemble is evaluated once on the held-out test set.

### 2.16. Explainability Analysis

Explanations were generated using Grad-CAM++ [37], an extension of Grad-CAM [38], applied to the final five-model EfficientNet-B4 ensemble. Grad-CAM++ uses spatially varying gradient-derived coefficients to weight convolutional feature maps, producing class-specific attribution maps. Gradients and activations were extracted from the final convolutional layer (conv_head) of each ensemble member.

Image preprocessing followed the classification pipeline, including EXIF orientation correction, aspect-preserving resizing to fit a 380 × 380-pixel canvas, Shades-of-Gray white balance (*p* = 6) applied before padding, centred zero-padding, and ImageNet normalisation. To preserve correspondence with the original clinical photographs, each resulting attribution map was cropped to remove the letterbox padding and resized to the original image dimensions using bilinear interpolation.

For each image, Grad-CAM++ maps were calculated independently for all five ensemble members. Each map was rectified and normalised to the range [0, 1] before aggregation. The ensemble attribution map was then obtained by averaging the five individual maps:

*H*(*x*, *y*) = (1/*K*) ∑*_k_ H_k_*(*x*, *y*), *K* = 5.

This procedure produces a combined attribution map representing the average spatial activation pattern across the ensemble.

These attribution maps were compared with reference masks marking the wound region: for each image, a rectangular box enclosing the wound was drawn by a single qualified cardiac surgeon before the Grad-CAM++ maps were viewed, so no reconciliation between raters was required.

Spatial agreement between the ensemble Grad-CAM++ attribution maps and expert-annotated wound regions was quantified using area-normalised relevance mass (ANRM), derived from relevance mass accuracy [39]. Let *H* ∈ ℝ ≥ 0^*N*^ denote the non-negative attribution map over *N* image pixels and *M* ∈ {0,1}^*N*^ the corresponding binary expert annotation, where *M_n_* = 1 identifies pixels inside the annotated wound region. The annotation area is *A* = ∑*_n_*_=1_^*N*^ *M_n_*, and its relative fraction of the image is *ρ* = *A*/*N*.

The fraction of total attribution mass contained within the annotated wound region is defined as

*q* = RMA(*H*,*M*) = (∑*_n_*_=1_^*N*^
*M_n_H_n_*)/(∑*_n_*_=1_^*N*^
*H_n_*). (1)

Because this fraction depends on the relative size of the annotation, we calculate the area-normalised relevance mass:

ANRM(*H*,*M*) = *q*/*ρ* = (*N* ∑*_n_*_=1_^*N*^
*M_n_H_n_*)/(*A* ∑*_n_*_=1_^*N*^
*H_n_*). (2)

ANRM = 1 corresponds to a spatially uniform attribution map, whereas ANRM > 1 indicates greater attribution concentration within the expert-annotated region than under uniform spatial distribution. Values below 1 indicate lower-than-uniform concentration. The calculations were performed on attribution maps restored to the dimensions of the original clinical photographs.

The analysis included 11 correctly classified Inflamed images and 4 false negatives analysed using separate Grad-CAM++ maps for the true class (Inflamed) and the predicted class (Good). ANRM was calculated for both classes to characterise their respective attribution distributions. These analyses assess spatial attribution alignment and do not establish causal reliance on the highlighted regions.

## 3. Results

### 3.1. Participants and Feasibility

**Figure 1:**
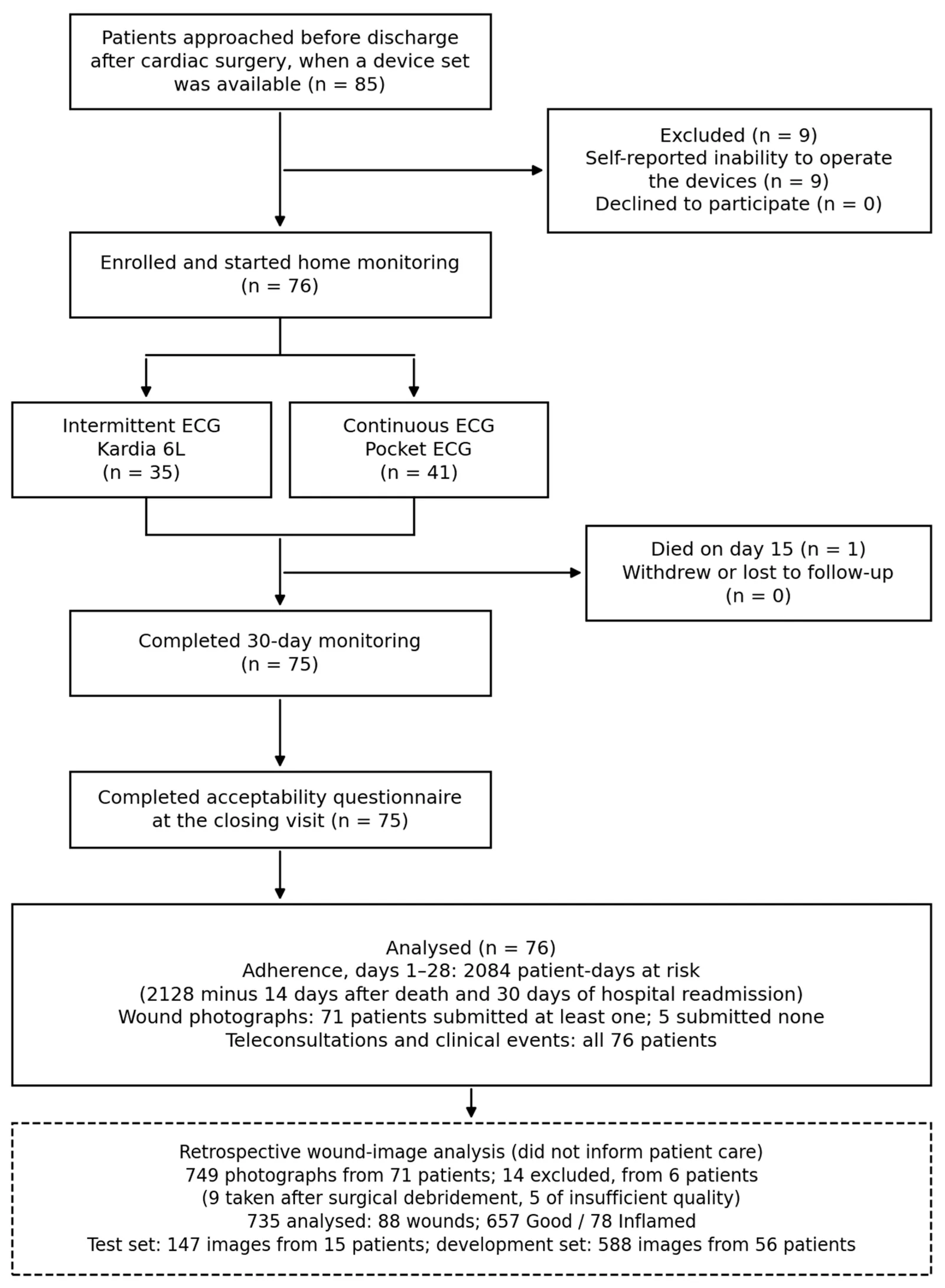
Participant flow. Six patients who were hospitalised during the programme paused monitoring and resumed it after discharge; they remained in the study. The retrospective wound image analysis included 735 of the 749 photographs received; the 14 excluded photographs came from 6 patients, and no patient or wound was excluded entirely.

Of 85 patients screened for eligibility, nine (11%) reported that they did not feel able to operate a camera-equipped smartphone and/or the monitoring equipment and were therefore ineligible; no eligible patient declined participation, and all 76 were enrolled (Figure 1). Seventy-five (99%) completed the 30-day programme, and one patient died on day 15. The mean patient age was 57.4 ± 11.9 years. The most frequently performed procedure was coronary artery bypass grafting (CABG) in 30 (39%) patients. The baseline characteristics of the study participants are presented in Table 3.

The mean total hospital stay of included patients, including preoperative days, was 9.7 ± 2.8 days. One patient (1.3%) died on the 15th day of the monitoring period. It was a 65-year old post-CABG female patient who died suddenly at home. She collapsed in the afternoon without preceding complaints, and the emergency team attempted resuscitation for 30 min without success. No post-mortem examination was performed and the cause of death was not established; according to her family, the emergency team suspected pulmonary embolism. She was taking aspirin 150 mg daily and was monitored with the Kardia 6L device. She received two teleconsultations. The first, on the 5th day of monitoring, was triggered by sinus tachycardia of 105 bpm in the ECG recording. The patient had her dose of bisoprolol increased from 2.5 mg to 5 mg once daily and was instructed to attend a visit in a cardiothoracic surgery outpatient clinic on the next day. The physical examination and blood sample tests performed during that visit did not reveal any abnormalities. The second, on the 9th day, was a supportive contact to reinforce adherence to treatment recommendations. From the 7th to 15th day of monitoring all recorded ECGs showed normal sinus rhythm in the range of 70 to 80 bpm; her last recording, made on the day of death, showed sinus rhythm at 70 bpm. She did not use any of the three communication channels throughout the 15 days monitoring period.

No device malfunctions attributable to patient handling occurred. Two brief Bluetooth-related interruptions occurred and were resolved remotely within 24 h.

### 3.2. Adherence

The mean monitoring time was 29.4 ± 1.0 days.

A total of 41 (54%) patients received the pocket ECG device and recorded a mean of 17.6 ± 4.3 h per patient/day of ECG samples. The remaining 35 (46%) patients with the Kardia 6L device provided a mean of 2.3 ± 0.4 ECG recordings per patient/day.

Over 2084 patient-days at risk, 4168 blood pressure/pulse and 2084 weight measurements were prescribed. Patients performed 3411 blood pressure/pulse measurements (adherence 81.8%) and 1694 weight measurements (81.3%), giving an overall adherence to the core measurement routine of 81.7% (5105/6252; 2.45 measurements per patient-day).

A total of 749 photographs of surgical wounds were received. Five patients did not send any photographs during the monitoring programme; they reported that taking and sending photographs was prohibitively challenging for them. Adherence to the photograph protocol was 65.7% (749/1140) across all 76 enrolled patients and 70.3% (749/1065) among the 71 patients who submitted at least one photograph. The adherence was better in the first half of the programme with 82.3% in the first week and 96.5% in the second week and decreased in the second half with 55.2% in the third week and 51.6% in the fourth week.

Details on the study adherence are presented in Table 4.

### 3.3. Acceptability

All 75 patients who completed the programme returned the questionnaire; five patients left at least one item unanswered. Each patient’s score was the mean of the items he or she answered, unanswered items being excluded; the mean score across patients was 6.3 ± 0.5 on the 7-point scale (File S1).

### 3.4. Teleconsultations and Clinical Events

**Figure 2:**
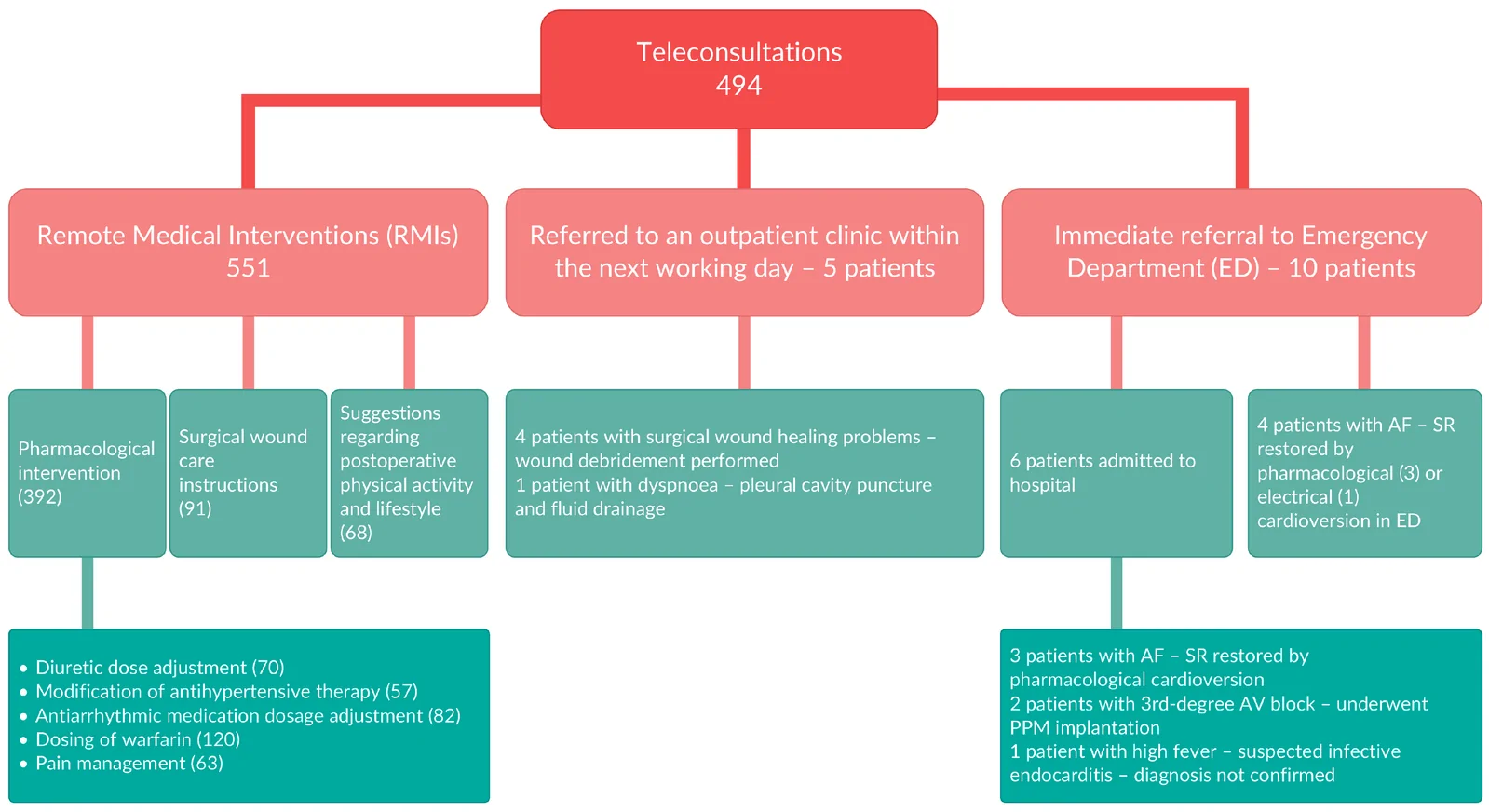
Flowchart for teleconsultations. Total RMIs (551) exceed teleconsultations (494) as patients may receive multiple interventions per call. Colours indicate the level of the flowchart: red, all teleconsultations; pink, type of action; green, resulting interventions and outcomes.

A total of 494 teleconsultations were held, and all 76 patients received at least one; the number per patient had a median of 5 (IQR 4–7; range 1–17). The 15 patients requiring INR control accounted for 228 teleconsultations (46%), with a median of 15 each (range 13–17), compared with a median of 5 (range 1–7) in the remaining 61 patients. During the teleconsultations, 551 RMIs were performed. The most frequent RMI was warfarin dose adjustment; the second most frequent was beta-blocker dose adjustment. The RMIs are summarised in Figure 2. Five patients were given the recommendation to visit the outpatient clinic on the next day, and 10 were urgently referred to the hospital emergency department. The most common indications for outpatient visits and emergency department referrals were problems with surgical wound healing and atrial fibrillation, respectively (Figure 2).

**Figure 3:**
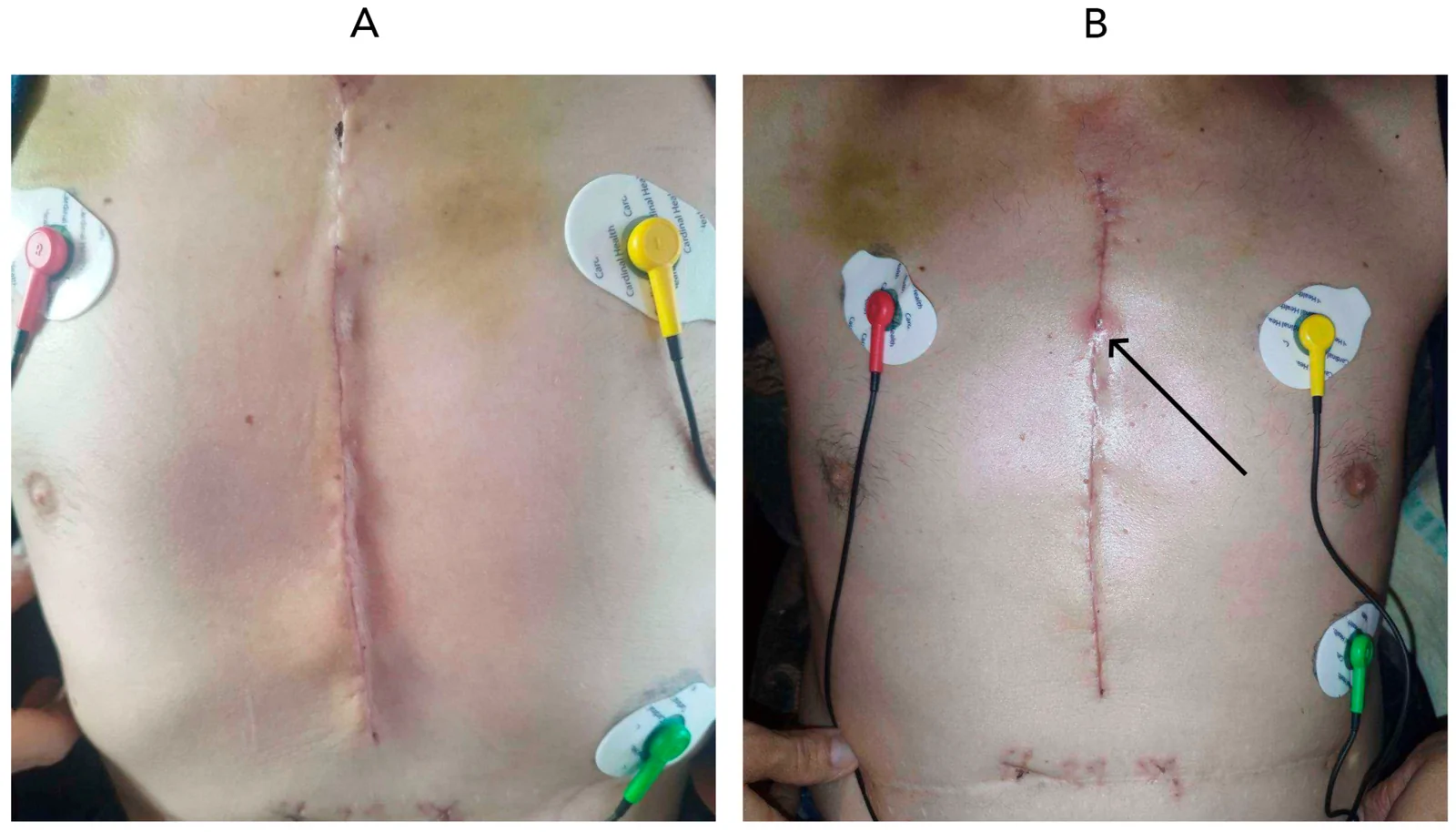
Surgical wound photographs of one of the patients. (**A**)—taken on the first day of the monitoring programme. (**B**)—taken on the 10th day of the monitoring programme—developing infection. Arrow pointing at the forming abscess. This wound was in the test set of the wound image classifier, which flagged it.

All four patients with surgical wound healing complications had wound cultures positive for bacterial growth and underwent surgical wound debridement, had silver wound dressings applied, and received antibiotics (Figure 3). All wounds were successfully treated in the ambulatory setting within 10 days at most.

Of the 10 patients referred to the hospital emergency department, four with AF were discharged home on the same day after restoring sinus rhythm with pharmacological treatment (3) or electrical cardioversion (1). Six patients were hospitalised during the monitoring programme. The most common indication for hospitalisation was AF. The second most frequent cause of hospitalisation was the third-degree atrioventricular block diagnosed in two patients after valvular surgery. Both patients underwent a permanent pacemaker implantation during the hospitalisation. The sixth admission was for high fever with suspected infective endocarditis, which was not confirmed. The mean hospitalisation time was 5 ± 2 days. Patients who were hospitalised suspended the monitoring and resumed it after discharge home.

Atrial fibrillation was confirmed in 18 episodes in 11 patients: 16 episodes in 9 of the 35 patients monitored with Kardia 6L and 2 episodes in 2 of the 41 patients monitored with pocket ECG. A total of 2 of these 11 patients had a history of paroxysmal AF before surgery; the remaining nine did not. Of the 11 patients, 7 were referred to the hospital emergency department and 4 had their pharmacological treatment modified during a teleconsultation, which included beta-blocker dose adjustment and the introduction of oral anticoagulant medication. In those patients, the sinus rhythm was restored within 48 h.

A total of 75 patients attended the outpatient consultation with the cardiologist on the last day of the monitoring programme. All patients were in sinus rhythm, healing of surgical wounds was normal, and echocardiography did not reveal any abnormalities.

Clinical events during the monitoring period are summarised in Table 5.

### 3.5. Clinician Workload

Daily review was performed during working hours, including weekends, by six clinicians (four cardiac surgeons and two cardiologists), so that transmitted data were reviewed within 24 h; arrhythmias detected on Kardia 6L recordings were additionally notified to the study staff by the 24/7 telemonitoring centre. Outside these review times, the study team provided no clinical cover, and patients were instructed to call emergency services in an emergency. Patient training at discharge was provided by staff of the telemedicine department. Per enrolled patient, the 30-day programme generated 6.5 teleconsultations and 7.3 remote medical interventions (approximately one teleconsultation every 4–5 monitored patient-days), together with 2.0 patient-initiated contacts (151 in total). The time spent on review and teleconsultations was not recorded.

### 3.6. AI-Assisted ECG Screening

**Figure 4:**
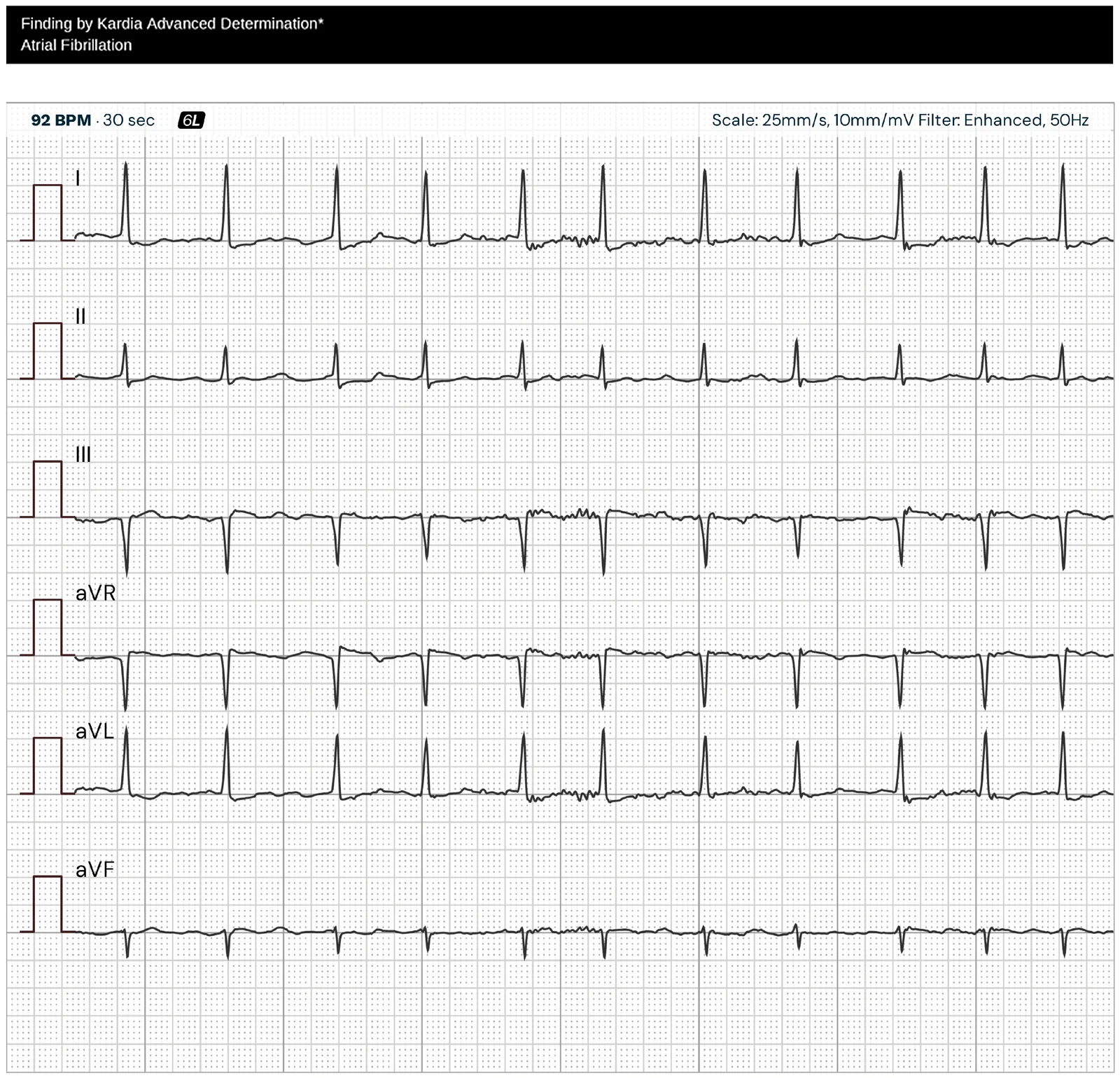
Atrial fibrillation flagged by the Kardia 6L algorithm and confirmed on clinician review. The banner shows the algorithm’s finding as displayed in the original report; patient details and the recording date have been removed.

All 18 AF episodes had been flagged by the device algorithms (Figure 4), and there was no disagreement between the reviewing staff and the cardiologist. Review of all Kardia 6L recordings found no AF in recordings that the algorithm had classified as sinus rhythm. Two further recordings flagged as AF by the Kardia 6L algorithm showed sinus rhythm with supraventricular premature beats. Monitoring exposure was a mean of 2.3 recordings per patient per day in the 35 patients with Kardia 6L and 17.6 h per patient per day in the 41 patients with pocket ECG.

### 3.7. Investigational Wound Image Classifier

Inter-rater agreement on the Inflamed label was high: observed agreement 97.8% (719/735), Cohen’s κ 0.89 (95% CI 0.83–0.94), positive specific agreement 89.9%, and Gwet’s AC1 0.97 (robust to the class imbalance). After adjudication of the 16 discordant images, 78 images were labelled Inflamed.

Table 6 reports 5-fold cross-validated validation metrics for the twelve screened configurations (six training-data configurations × two backbones), used for selection, together with the final Optuna-tuned model (EfficientNet-B4+RedScar+SurgWound), for which we give both its cross-validated validation and held-out test performance of the ensemble of its five fold-specific models. Inflamed is the positive class.

### 3.8. Screening

**Figure 5:**
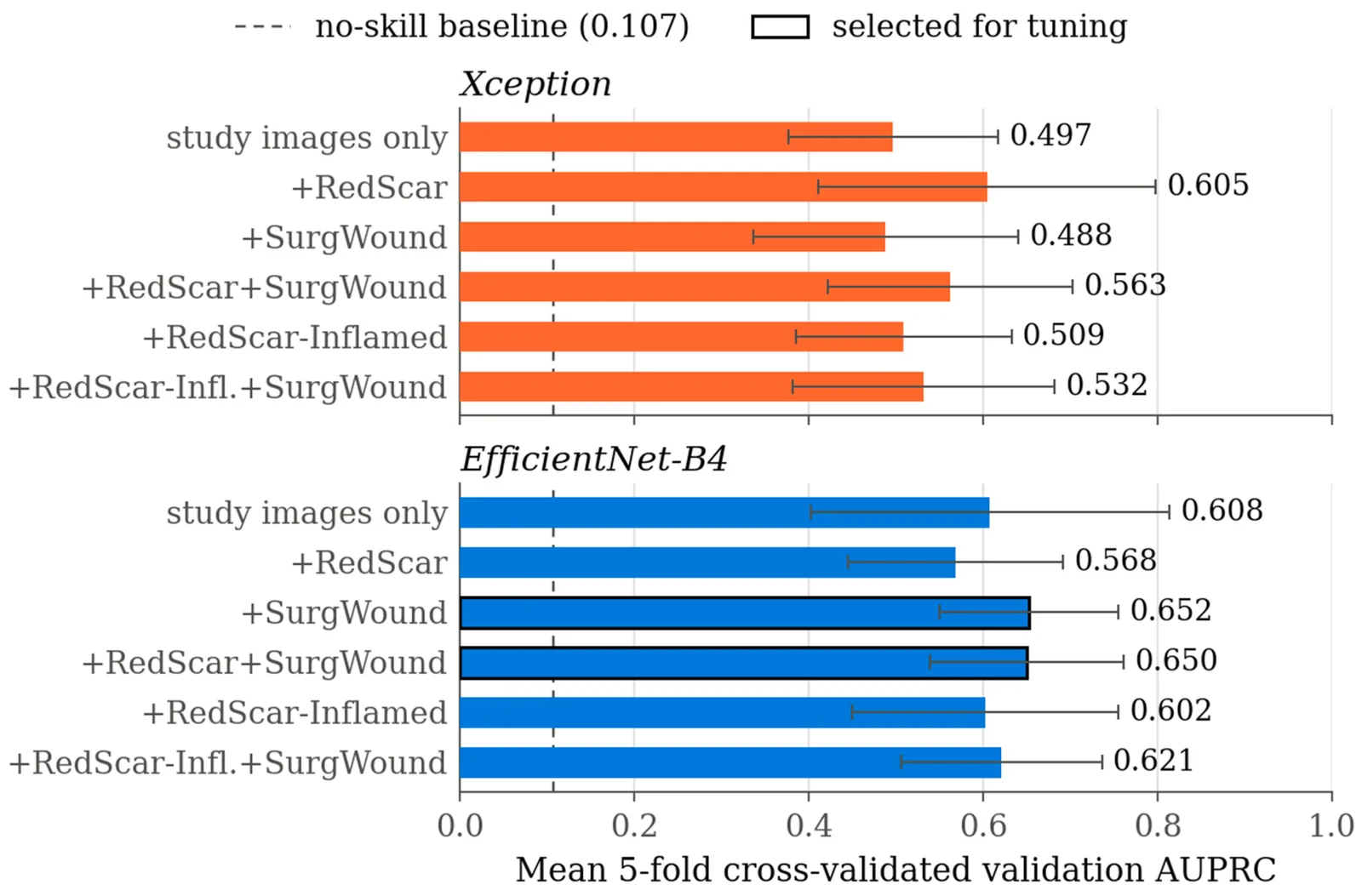
Mean 5-fold cross-validated validation AUPRC for the twelve screened configurations (two backbones × six training-data configurations), with error bars showing the standard deviation across folds. The dashed line marks the no-skill baseline given by the positive prevalence of the validation folds (0.107). EfficientNet-B4+SurgWound and EfficientNet-B4+RedScar+SurgWound (outlined) are the configurations carried forward to hyperparameter optimisation. Orange bars, Xception; blue bars, EfficientNet-B4.

Ranked by mean cross-validated validation AUPRC, the EfficientNet-B4 configurations lead: B4+SurgWound (0.652) and B4+RedScar+SurgWound (0.650) are the top two, followed by B4+RedScar-Infl.+SurgWound (0.621), and the highest validation ROC-AUC in the screen is also B4’s (0.888, B4+RedScar+SurgWound), though B4 does not lead Xception uniformly: with +RedScar, Xception is ahead (0.605 vs. 0.568). The differences among the leading configurations are, however, within one cross-validation standard deviation (≈0.10–0.21 across the twelve configurations), so the ranking is a lean rather than a decisive ordering. One qualitative pattern is apparent: the three top-ranked configurations all add SurgWound to the training data. We therefore carry the two top-ranked configurations, EfficientNet-B4+SurgWound and EfficientNet-B4+RedScar+SurgWound, whose means differ by only 0.002, forward to hyperparameter optimisation. Figure 5 shows the full ranking: every configuration clears the no-skill baseline of 0.107 by a wide margin, but the twelve means span only 0.488–0.652 and every ±1 SD interval overlaps those of the selected configurations.

### 3.9. Effect of Hyperparameter Optimisation

**Figure 6:**
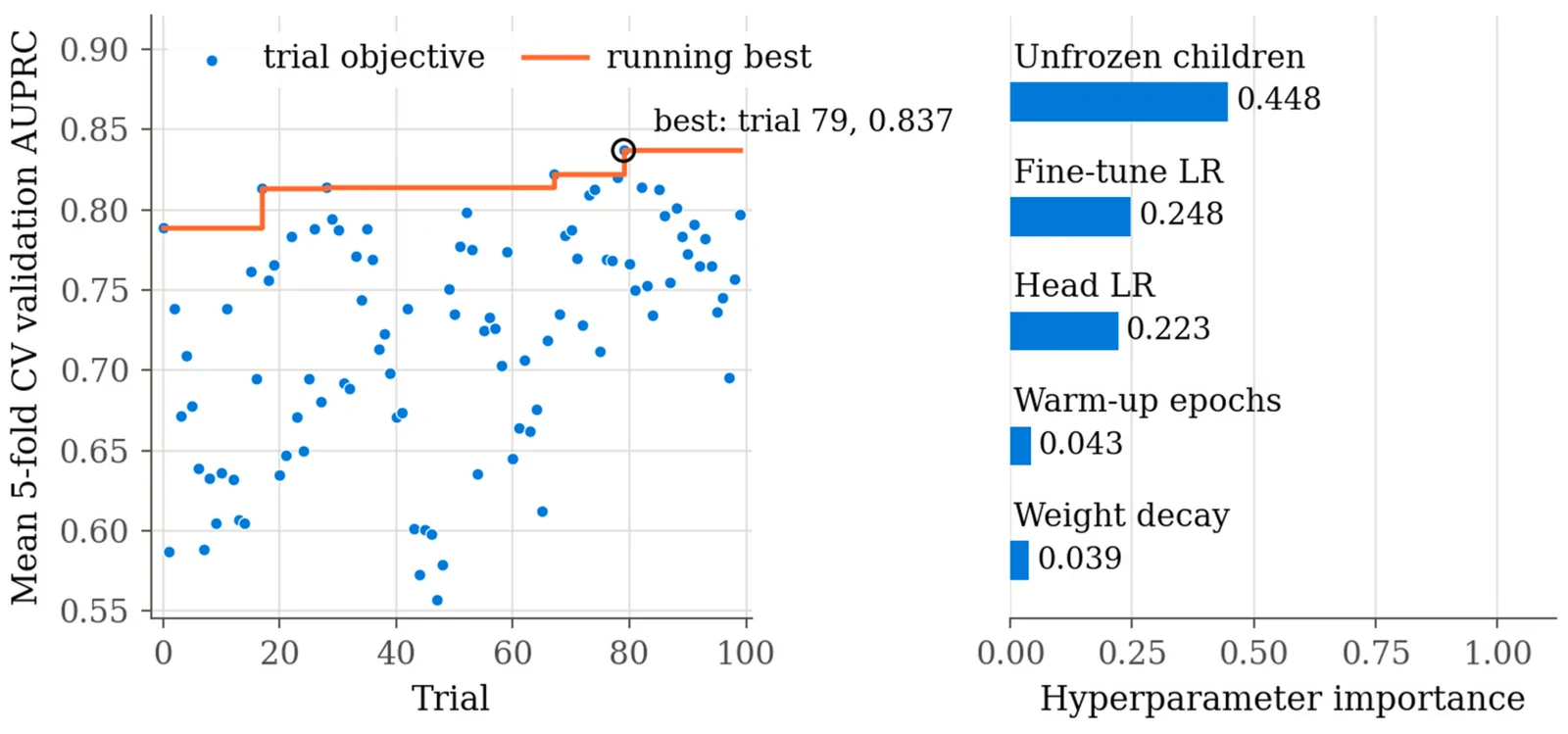
Hyperparameter optimisation of EfficientNet-B4+RedScar+SurgWound. (**Left**): Optimisation history over the 100 TPE trials (per-trial mean 5-fold cross-validated validation AUPRC and running best). (**Right**): fANOVA hyperparameter importances for the same study, covering the five hyperparameters sampled in every trial; batch size was sampled within each unfreeze regime separately and is therefore not comparable across trials.

Both configurations were tuned. For the best-trial hyperparameter settings, a fresh 5-fold cross-validation yielded a validation AUPRC result of 0.801 for EfficientNet-B4+SurgWound and 0.771 for EfficientNet-B4+RedScar+SurgWound. Because the two tuned configurations could not be separated on the primary selection metric (a difference smaller than one fold-to-fold standard deviation, 0.097 and 0.153), the choice between them was made on cross-validated operating-point metrics relevant to triage, on which EfficientNet-B4+RedScar+SurgWound performed better (recall 0.643 vs. 0.566; balanced accuracy 0.812 vs. 0.770; Inflamed-F_1_ 0.697 vs. 0.626). This decision was based on validation data only, before the test set was evaluated, and the results below refer to this model. Tuning improved the results substantially over its default hyperparameter screen. The decisive change was unfreezing the backbone: the default fine-tuned only the head (unfreeze = 4, body frozen), whereas 87 of the 100 trials unfroze at least the full body (unfreeze = 5 or 7), and the best AUPRC trial unfroze the entire backbone (unfreeze = 7). Table 7 contrasts the default and tuned hyperparameters. Figure 6 summarises the course of the search: the best configuration was reached in trial 79 (mean CV validation AUPRC 0.837), and an fANOVA analysis attributes the largest share of the objective’s variance to the number of unfrozen children (0.448), ahead of the fine-tuning (0.248) and head (0.223) learning rates. Under 5-fold cross-validation, this lifts validation AUPRC from 0.650 (default, head-only) to 0.771, and the final model, the ensemble of the five fold-specific models, reaches test AUPRC 0.747, ROC-AUC 0.927, balanced accuracy 0.852, recall 0.733 (11/15), and Inflamed-F_1_ 0.733. The tuned model’s cross-validated validation AUPRC and the held-out test AUPRC of its ensemble are similar (0.771 vs. 0.747). (The Optuna selection AUPRC, 0.837, is the maximum over 100 trials and is therefore optimistically biased; the 0.771 reported here is a fresh cross-validated estimate at the selected hyperparameters.)

**Figure 7:**
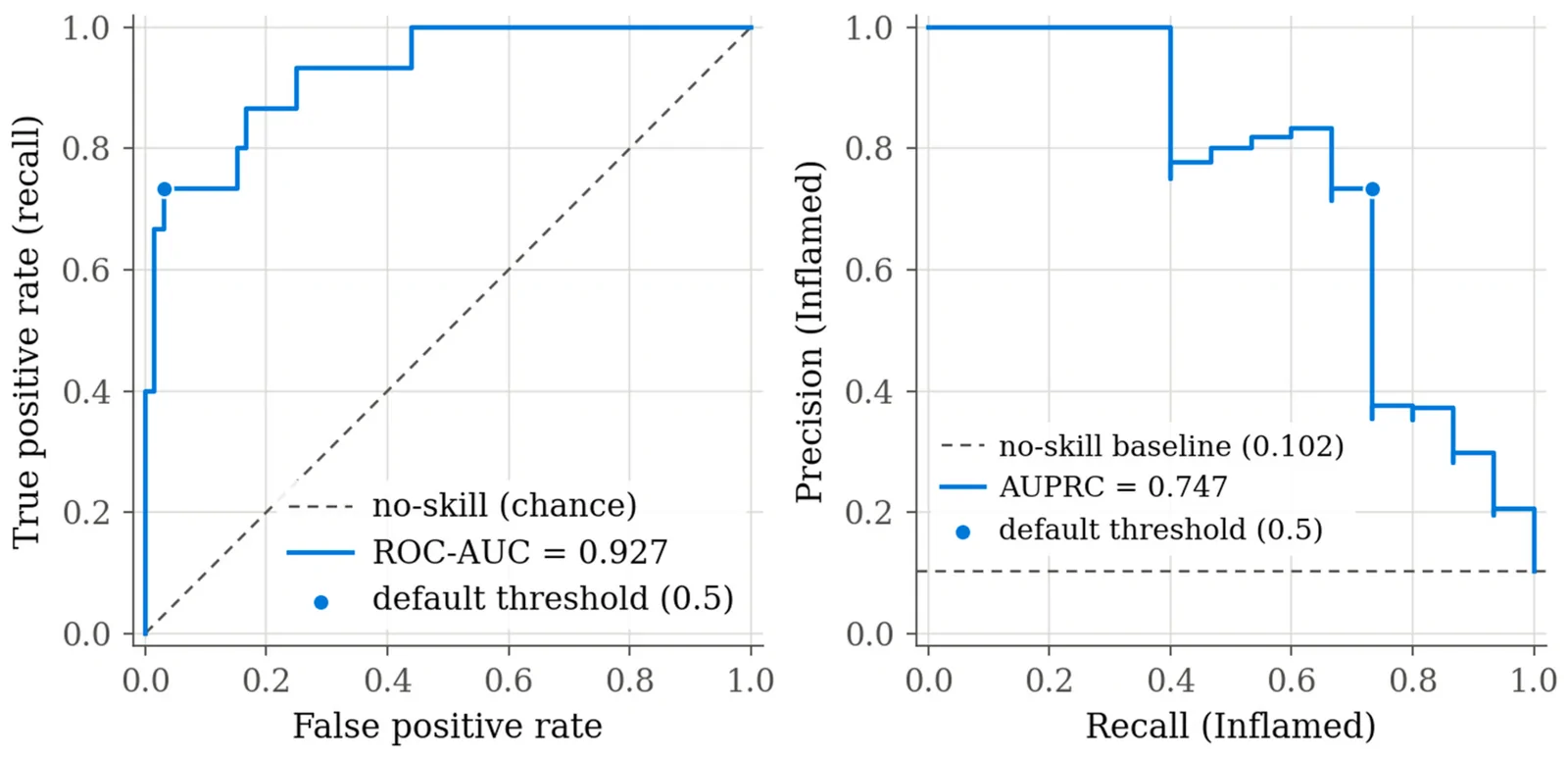
Receiver operating characteristic (**left**) and precision–recall (**right**) curves of the tuned EfficientNet-B4+RedScar+SurgWound ensemble on the held-out test set (147 images, 15 Inflamed). ROC-AUC 0.927; AUPRC 0.747 against a no-skill baseline of ≈0.10. The marked point is the operating point at the default decision threshold, i.e., the one described by Figure 8.

**Figure 8:**
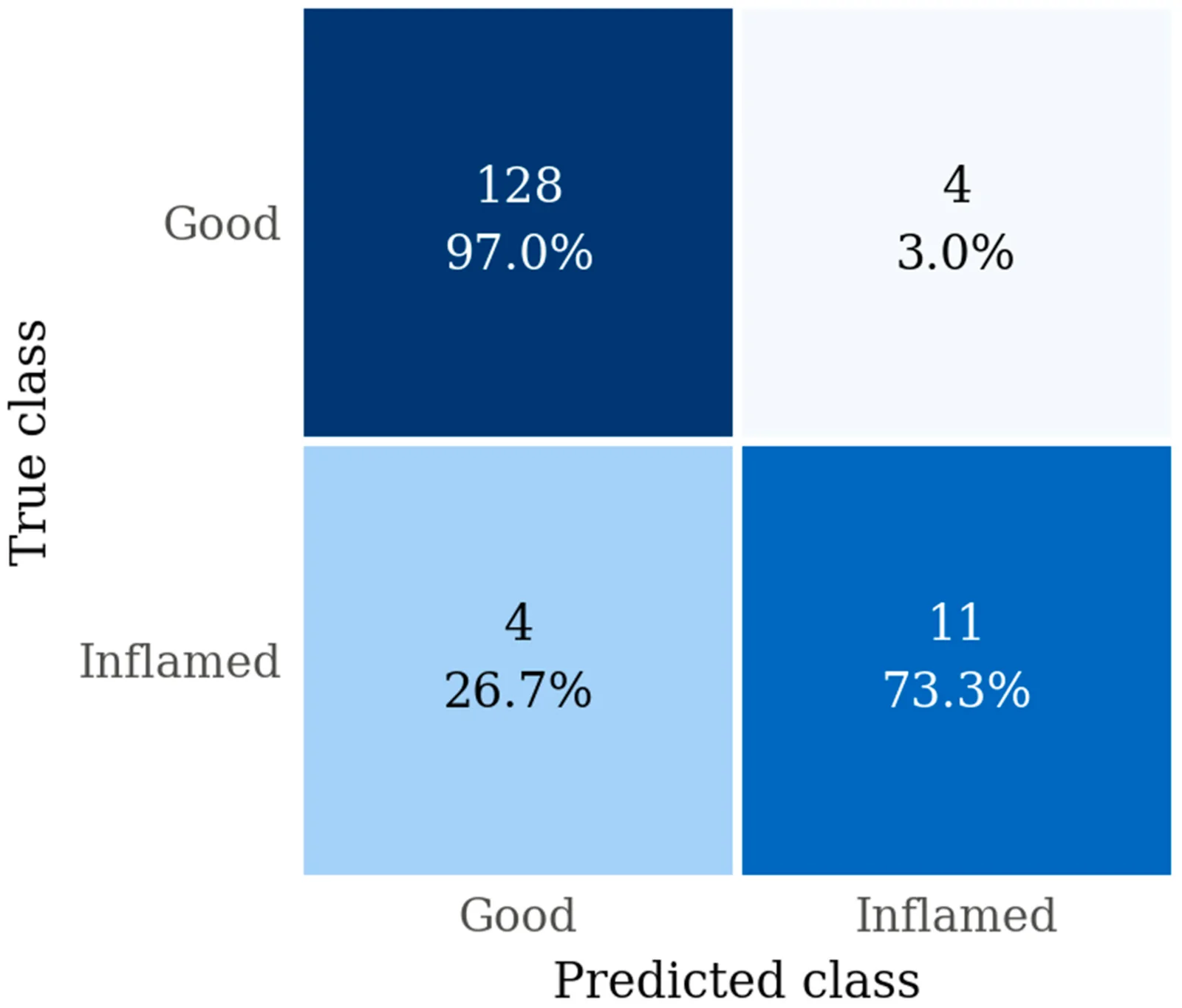
Confusion matrix of the tuned EfficientNet-B4+RedScar+SurgWound ensemble on the held-out test set at the default decision threshold (147 images, 15 Inflamed): recall 0.733 (11/15), precision 0.733, and overall accuracy 0.946. Cells give the image count and the percentage of their true class and are shaded by that percentage (darker blue indicates a higher percentage).

The discrimination of the final model on the held-out test set is shown in Figure 7: the ROC and precision–recall curves give ROC-AUC 0.927 and AUPRC 0.747 against a no-skill baseline of 0.102. Figure 8 resolves the same operating point into counts, where 11 of the 15 Inflamed images are detected, at the cost of four false positives among the 132 Good images.

At the wound level, the 15 inflamed test images arise from six distinct wounds of six patients; counting a wound as detected when at least one of its images was flagged, the model detected five of the six inflamed wounds (wound-level sensitivity 5/6)—a lenient rule that reflects the intended use, in which flagging any image of a wound brings it to clinician attention. Because each of the six patients has a single inflamed wound, the wound-level sensitivity is also the patient-level sensitivity (5/6). The single missed wound was a sternotomy wound, none of whose four test images were flagged. Of the four patients with confirmed wound complications, one was allocated to the test set by the random patient-level split, and the classifier flagged this wound, which is shown in Figure 3; the missed sternotomy wound did not meet the definition of a confirmed complication. The other three patients with confirmed complications were in the development set and could therefore not be used to evaluate the classifier. All photographs of these four patients that entered the analysis were taken before surgical debridement (Methods); their timing relative to first clinical recognition or the start of antibiotic treatment was not analysed, so some of these photographs may postdate clinical recognition.

### 3.10. Explainability

**Figure 9:**
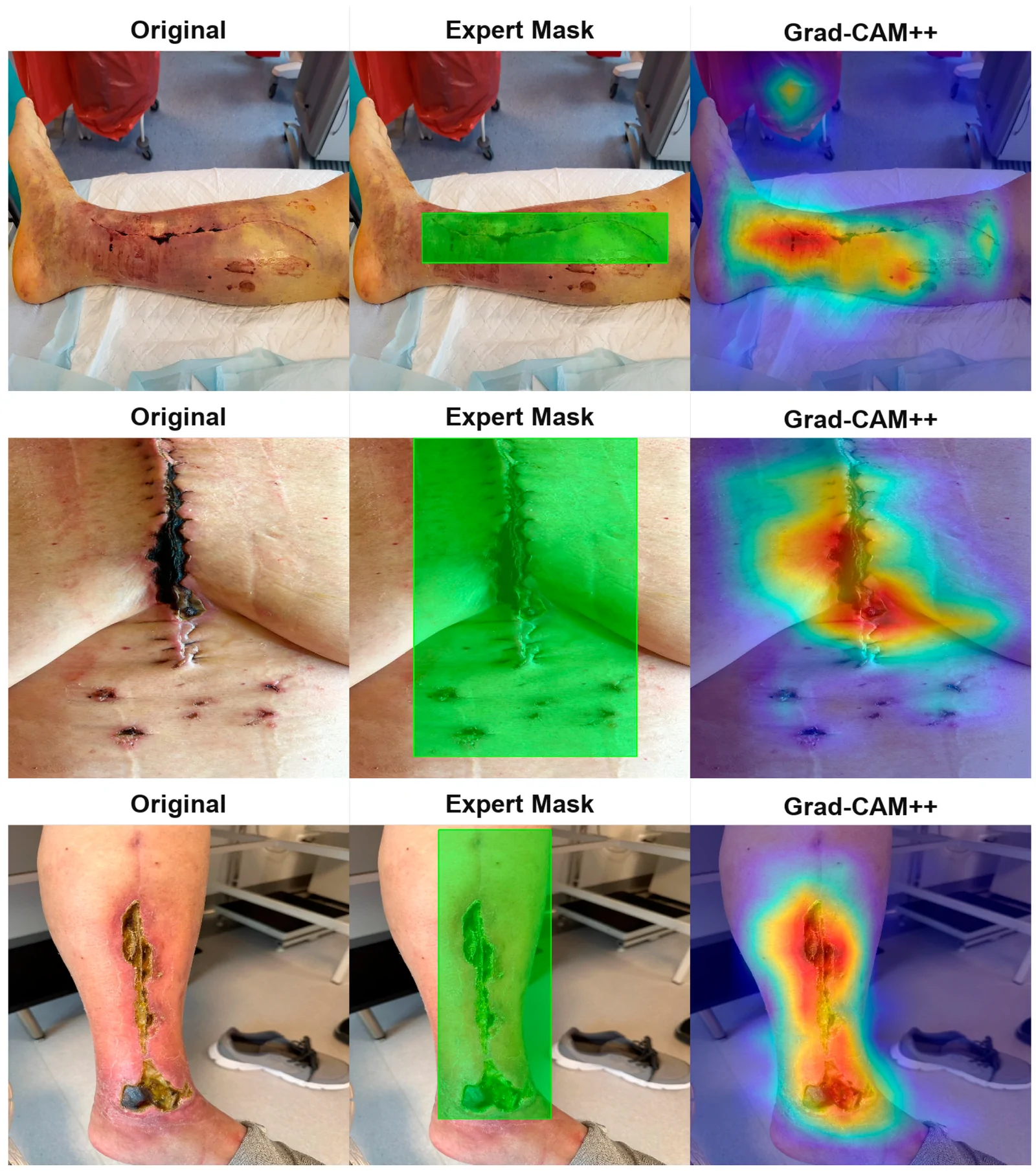

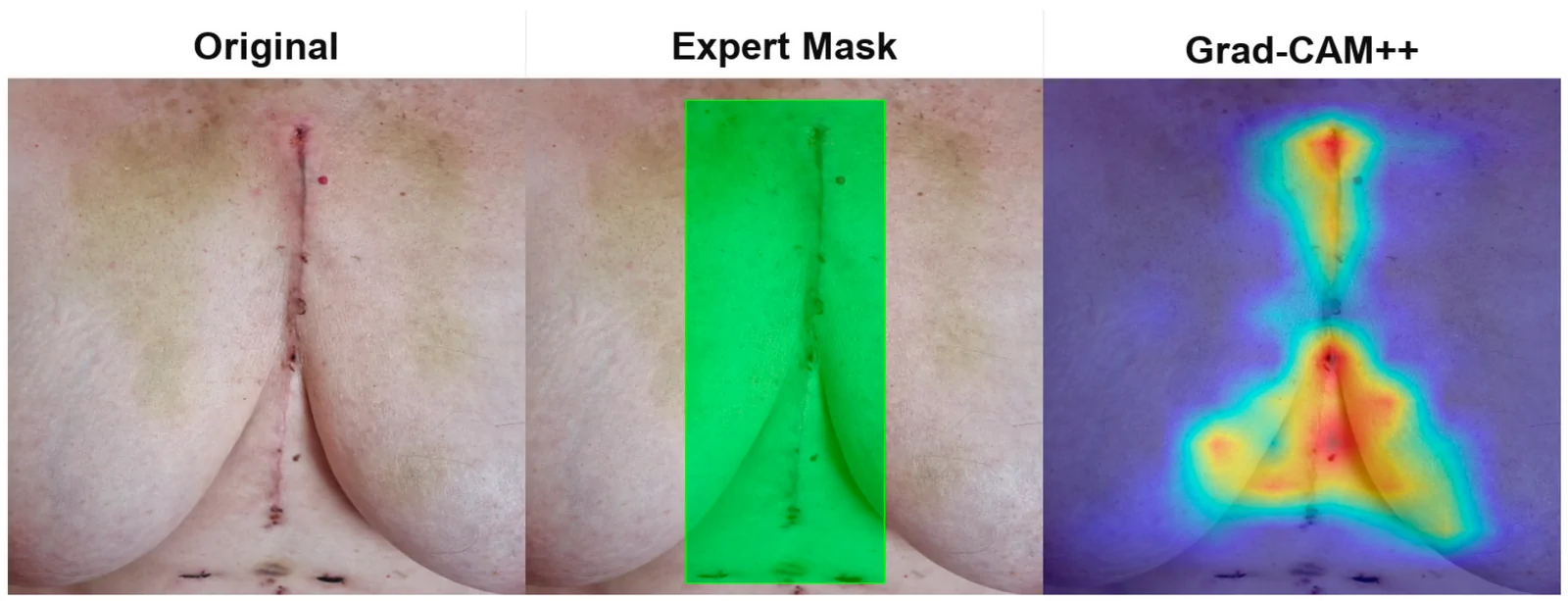
Grad-CAM++ explanations for three inflamed test images that were correctly classified and one inflamed image misclassified as Good. Each row shows, from left to right, the original photograph, the expert-annotated wound region, and the Grad-CAM++ heatmap. Rows, top to bottom: Leg 9-b, Sternotomy 10 and Leg 2-b (correctly classified as Inflamed), and Sternotomy 5-d (misclassified as Good; Grad-CAM++ map for the true class, Inflamed). The expert-annotated wound region is shown as a green box; Grad-CAM++ heatmaps use the Jet colour map, from blue (low) to red (high attribution).

Table 8 presents the area-normalised relevance mass (ANRM) of the ensemble Grad-CAM++ attribution maps for the 11 correctly classified Inflamed test images. ANRM exceeded the uniform attribution reference value of 1 in every image, ranging from 1.40 to 4.71, with a mean of 3.08 and a median of 3.01. Thus, the attribution maps showed consistently higher concentration within expert-annotated wound regions than a spatially uniform attribution map. Nine images reached ANRM above 2, and six exceeded 3. The two highest values (4.71 and 4.48) came from repeat photographs of a single wound (Leg 1), and the lowest, 1.40, was still above the uniform reference. Visual inspection agreed with the numbers: the strongest activations generally followed the wound or its immediate surroundings (Figure 9, first three rows).

The four false-negative images, all from a single patient, were examined separately using Grad-CAM++ attribution maps calculated for both the true (Inflamed) and predicted (Good) classes. The mean ANRM was 2.00 for the Inflamed class and 1.86 for the Good class (see Table 9). In both cases, attribution concentration within the expert-annotated wound regions exceeded the uniform reference, despite the model’s incorrect classification (Figure 9, last row).

## 4. Discussion

### Main Findings

This prospective study demonstrates the feasibility of a remote home monitoring programme with AI-assisted ECG screening in patients discharged home after cardiac surgery. The results indicate that such a programme is feasible in a real-world cardiac surgery setting, with acceptable protocol adherence and few technical constraints. Complications identified during daily review were managed by remote medical interventions (RMIs) or in the ambulatory setting. A total of 6 of 76 patients (7.9%) were admitted to hospital during the monitoring period; in the absence of a control group, this figure describes the cohort and cannot be compared with published readmission rates.

Feasibility was supported across several aspects of the monitoring programme. The principal barrier to participation was patients’ perceived ability to use the technology: 9 of 85 screened patients (11%) reported that they could not operate the smartphone or monitoring equipment, whereas all patients who felt able to do so agreed to take part. All but one patient (the one who died) completed the 30-day monitoring period, with 82% adherence to the core measurement routine (twice-daily blood pressure/pulse and daily weight). Technical issues were rare and minor (only two brief Bluetooth interruptions, both resolved remotely within 24 h), and photograph adherence was 65.7% across all enrolled patients and 70.3% among the 71 who submitted at least one photograph.

Photo submission showed the steepest adherence decline of any measurement, falling from the first to the second half of the programme. This likely reflects that wound photography was the most technically demanding task, requiring correct framing and transmission on top of the routine device measurements; for some patients—plausibly older participants with more limited smartphone literacy—it may have depended on a family member to capture and send the image, adding a third-party step that the other measurements did not require; caregiver involvement was not recorded. A future iteration could mitigate this with weekly SMS reminders reinforcing the importance of the wound photographs, alongside a simplified submission interface. In routine use, photographs that cannot be assessed should never be classified silently: an automated image-quality check at submission could ask the patient to retake an unusable photograph, and repeated failures would be passed to a clinician for review.

Teleconsultation workload was concentrated in patients on vitamin K antagonists: one fifth of the cohort accounted for almost half of all teleconsultations, because repeated dose adjustment required 13–17 contacts each. Structured anticoagulation management would be the first target for reducing clinician workload.

The classifier’s results are best read as a hierarchy of three claims of decreasing evidential strength. First and most firmly, the model reproduces the clinicians’ visual inflamed/not-inflamed judgment of a photograph: this is the task it was trained and tested for, the reference reading was itself highly reproducible (Cohen’s κ 0.89 between two independent clinicians), and on the held-out set, the model matched the consensus label for 11 of 15 images and 5 of 6 wounds. Second, and more tentatively, it identifies inflamed wounds—a clinical state inferred through the same visual channel and therefore bounded by the reliability of that channel. Third, and most tentatively, it may flag wounds that ultimately require intervention—the only claim resting on a reference standard external to the photograph. Under the patient-level split, however, only one of the four confirmed complications fell in the test set, so this claim could be examined in a single case, which the classifier flagged. The tool is therefore best positioned for triage: it prioritises images for clinician review instead of diagnosing infection.

Two design choices were decisive for the classifier performance we obtained. First, model screening and hyperparameter optimisation were guided by the mean 5-fold cross-validated AUPRC of the Inflamed class. The final choice between the two tuned configurations, whose AUPRC values differed by less than one fold-to-fold standard deviation, additionally considered cross-validated recall, balanced accuracy, and Inflamed-class F_1_. All selection decisions were based on validation data alone. Second, adapting the pretrained representation rather than only the classification head—the tuned model unfreezes the entire EfficientNet-B4 backbone—is the largest single contributor to the gain over the untuned screen (validation AUPRC 0.650 to 0.771). The resulting operating point, recall 0.733 at precision 0.733, flagged five of the six inflamed test wounds while keeping the number of images escalated for clinician review small; with confidence intervals this wide (recall 0.333–1.000), however, it indicates the profile a triage tool would need rather than establishing it.

The explainability analysis showed spatial alignment between ensemble Grad-CAM++ attribution maps and expert-annotated wound regions. ANRM exceeded the uniform reference in all 11 correctly classified Inflamed images (mean 3.08), and even the four false-negative images showed above-uniform concentration of attribution within the marked region for both the Inflamed and Good classes. These findings show that spatially aligned explanations can accompany both correct and incorrect predictions. However, such alignment alone does not prove causal reliance on the highlighted regions or exclude the influence of background features.

## 5. Limitations

Study limitations include its single-centre design, lack of a randomised control group, and modest sample size (*n* = 76). Nevertheless, such a sample size is typical and appropriate for a prospective evaluation of feasibility (recruitment, retention, adherence, technical performance) and acceptability [40]. This approach is well established and widely accepted in digital health and remote monitoring research, where smaller cohorts have repeatedly provided meaningful insights into real-world implementation [41]. Because the study was single-arm, it shows that the programme detected abnormalities and triggered clinical actions, but it cannot establish that the programme prevented admissions, reduced complications or readmissions, detected complications earlier than standard care, or improved outcomes; these questions require a randomised comparison. The cohort was also lower-risk than the populations behind the 12–20% readmission rates reported in the literature [6,42], so the observed admission rate is not evidence of benefit. The one death in the cohort was sudden and occurred on a day when the patient’s ECG recording had been normal. Intermittent 30 s recordings cannot capture an abrupt event between recordings, and the programme was not designed as, and should not be regarded as, an emergency surveillance system.

Because the ability to operate a smartphone and the monitoring devices was an eligibility criterion, the cohort was selected for digital capability. Its adherence and acceptability therefore describe patients able to use the technology and are likely to overstate what would be achieved in an unselected cardiac surgical population, particularly among older patients or those without family support. Capability was self-reported, so some patients who declared themselves unable may have been reluctant rather than incapable. In addition, recruitment depended on the availability of monitoring sets rather than being consecutive, which may have introduced selection bias, and feasibility outcomes were reported descriptively rather than against prespecified success thresholds.

Escalation decisions depended on the judgment of the reviewing clinician rather than on prespecified rules, which limits the reproducibility of the intervention; a future protocol should define explicit escalation criteria. Staff time was not measured, so the study establishes the technical feasibility of the programme more firmly than its scalability; time and motion data and a cost analysis are priorities for the next study. Acceptability was measured with a study-specific questionnaire that has not been validated; item-level results and internal consistency are not reported, and scores from a cohort selected for digital capability may overstate acceptability in an unselected population.

The ECG findings describe detection yield, not diagnostic accuracy. Every Kardia 6L recording was reviewed by staff, but pocket ECG was reviewed, as in standard ambulatory ECG monitoring, through algorithm-generated events and daily reports rather than beat by beat, so AF that the algorithm did not detect could have gone unrecognised; events transmitted outside review hours were reviewed at the next daily review, within 24 h. The two devices also differed in monitoring mode, review pathway, and the patients assigned to them, so the difference in the number of AF episodes between them does not reflect device performance.

A further limitation concerns the evaluation of the wound classifier: the held-out test set contains only 15 positive (Inflamed) images from six patients, so the threshold-dependent metrics (recall, precision, F_1_, balanced accuracy) change in coarse steps, and the threshold-free AUPRC and ROC-AUC are the more reliable indicators. The 95% confidence intervals around the test estimates are correspondingly wide (for example, AUPRC 0.402–1.000 and sensitivity 33.3–100%), so the classifier results should be regarded as preliminary feasibility estimates rather than robust estimates of diagnostic performance. For the untuned screen, cross-validated validation AUPRC spanned ≈0.49–0.65; the tuned model reached 0.771 under cross-validation and its ensemble 0.747 on the test set. Confirmation on a larger set of positives, and external validation, therefore remain warranted before fixing a single operating point for clinical use. A second, related limitation is that, although the test set and all cross-validation folds are patient-disjoint, so that the test figures describe performance on patients the classifier has never seen, all images come from a single centre and were labelled by the same readers; validation on an external, multicentre dataset is the necessary next step. A third limitation concerns the external training data. Their labels derive from an infection risk annotation (SurgWound) and an infection flag (RedScar) rather than from the clinicians’ Southampton grading of visible inflammation, and the photographs differ from ours in patient population, wound types, and acquisition conditions; they therefore add label noise and a domain shift to the training data. Because the external images never enter validation or testing, this does not change what the reported metrics measure, but it may limit the benefits of enrichment.

Three further cautions bear on interpretation. First, the primary reference standard is image-derived: the Inflamed label is a consensus visual reading of the same photograph the model sees, so the primary metrics measure concordance with expert reading, not detection of infection. Only the confirmed complication subset rests on a standard independent of the image. Second, ascertainment of those complications is partly entangled with the photograph’s appearance, since a wound was escalated partly because it looked concerning; that subset therefore rests on more than the image (debridement, culture, and the day-30 review) but is not fully independent of it. Third, only one of the four confirmed complications fell in the test set; it was flagged, but a single case cannot show how reliably the classifier identifies wounds that require intervention. Moreover, because the classifier was applied retrospectively, flagging this wound shows that it recognised it, not that it would have identified it earlier than the clinicians did.

Although these results are promising for a feasibility study, they remain constrained by the size of the available dataset. It is a well-established phenomenon in the deep learning era that model performance on vision tasks scales logarithmically with the volume of training data [43]. We posit that the current metrics represent a baseline which may improve as the remote monitoring programme expands and the dataset grows. Our screening experiments point in the same direction: the three best-ranked configurations all enlarged the training set with external SurgWound images. Additional annotated positive cases are likely to remain the limiting resource, as the positive class accounts for only 78 of the 735 study images.

Two cautions apply to the explainability results. Spatial agreement with an expert mask is evidence of alignment, not proof of causal faithfulness. And because inflamed cases are few, several of the 11 images come from the same wound photographed on different dates, so the 11 ANRM values are not independent.

Finally, in accordance with the SAGER guidelines, we note that the cohort was predominantly male (55/76, 72%) and that no sex- or gender-stratified analysis of monitoring adherence or classifier performance was undertaken, because the number of events in each subgroup was too small to support such a comparison.

Comparison with Previous Studies

Our findings extend and complement previous work on post-cardiac surgery remote monitoring. The Box 2.0 study prospectively enrolled 365 patients for scheduled self-measurement of blood pressure, temperature, weight, and ECG and, compared with 365 historical controls, detected postoperative atrial fibrillation more often over three months (16.7% vs. 6.8%) [44,45]. The SEARCH-AF trial randomised 336 patients at high stroke risk to 30 days of continuous patch-based rhythm monitoring or usual care and detected AF lasting at least 6 min in 19.6% vs. 1.7% [46]. Both studies differ from ours in ways that preclude direct comparison of AF rates: each had a comparator group and AF detection as its primary end point; SEARCH-AF enrolled selected high-risk patients without a history of AF and used a 6 min threshold, whereas 18% of our patients had a history of paroxysmal AF; and Box 2.0 monitored for three months. Our study was single-arm and descriptive; what distinguishes it is daily clinician review with direct teleconsultation across several parameters, AI-assisted ECG screening, and a retrospective evaluation of an investigational wound image classifier.

Atilgan et al. followed 2340 patients for up to one year [16]. However, in their large cohort, patients averaged just 0.12 blood pressure measurements per day and a mere 0.03 live ECG/Holter recordings per day—reflecting relatively sparse data capture that may limit proactive detection of subtle deteriorations. By contrast, our protocol focused on the 30-day monitoring window, specifically targeting the highest-risk period for readmissions. It mandated twice-daily blood pressure and pulse assessments (achieving 1.6 measurements per patient-day at 82% adherence) alongside thrice-daily 30 s ECG recordings (or continuous wear via the pocket ECG device). This denser data stream allowed daily review of trends; whether it leads to earlier intervention than sparser monitoring would have to be tested in a comparative study. Our patient sample, while smaller, was drawn from a Polish population, adding geographic and healthcare-system specificity to the evidence base.

A distinctive feature of this study is its retrospective evaluation of an AI-based classifier on patient-submitted wound photographs, a novel component that sets it apart from prior remote monitoring efforts in cardiac surgery: patients themselves supplied the photographs, on alternate days, throughout the 30-day window. More broadly, what distinguishes the present programme is not any single component but the combination of three: the density of the data stream and its daily review by a clinician; three possible teleconsultation outcomes, each quantified; and two algorithmic roles kept explicitly apart—the off-the-shelf ECG algorithms that screened recordings as they arrived and informed patient management, and a purpose-built wound classifier developed and evaluated retrospectively as a proof of concept.

The closest precedent is the WISDOM AI study, which flagged surgical wound photographs requiring priority clinician review [20]. Its scale was far larger (37,974 training and 3634 test images) and its split was patient-level, with one image per patient; ours was also patient-level but included several images per patient, which the patient-clustered bootstrap confidence intervals take into account. Its reference standard nonetheless matched ours—two specialist nurses with a third adjudicator, scoring concordance with expert visual assessment rather than infection—and it reached 89% sensitivity (95% CI 87–91%); our image-level sensitivity, 73.3% (95% CI 33.3–100%), was lower and far less precise. Our cohort is smaller but adds prospective in-workflow collection in cardiac surgery, a quantified reference-standard reliability (κ 0.89), and a quantitative explainability analysis, which WISDOM left to future work. Both frame the tool as reproducing, not replacing, clinician judgment.

A larger multicentre study from nine Mayo Clinic hospitals addressed the harder task, using 20,895 patient portal images from 6060 patients and a two-stage pipeline (incision then infection detection) referenced against independently ascertained National Surgical Quality Improvement Program (NSQIP) infection outcomes rather than image appearance [21]. Incision detection reached 0.94 accuracy but infection detection only 0.81 AUC at 0.53 recall, confirming that predicting a verified infection from a photograph is far harder than reproducing a clinician’s visual impression—the task our own metrics address. NSQIP excludes cardiac surgery, so that cohort contained none; its scale and independent reference standard define the benchmark that a future external validation of our approach should aim to meet.

Atrial fibrillation was identified in eleven of our patients during monitoring. In such patients, cardiac rehabilitation combines clinical stabilisation, optimisation of therapy, structured exercise, and secondary prevention [47]; a monitoring programme of the kind described here could provide a bridge from discharge to rehabilitation, and integrating the two is a natural next step.

## 6. Conclusions

The wound image classification results suggest that an image recognition-based, human-in-the-loop triage tool could be integrated into routine post-discharge monitoring, once validated on a larger, multicentre dataset. In practice, the system would assign each photo a simple risk signal to prioritise a small subset for rapid clinician review, while keeping all images available for standard oversight. Additionally, a small random sample of routine-appearing images would also be reviewed to audit for missed issues and detect performance drift. Verified outcomes from flagged and sampled reviews would be fed back to build a growing, clinically validated dataset, enabling gradual refinement while aiming to avoid universal manual review; whether such a workflow reduces clinician workload remains to be tested.

Self-monitoring programmes such as this one may also increase patients’ awareness of their health status and engage them more actively in their own recovery; our questionnaire did not measure this directly, and it remains a hypothesis for future studies.

In conclusion, this remote monitoring programme, with AI-assisted ECG screening and a candidate AI layer for wound image triage, offers a framework for closer follow-up in the first 30 days after cardiac surgery. Multicentre randomised studies are warranted to quantify impacts on readmissions, costs, and long-term outcomes.

## Figures and Tables

**Table 1:** Training hyperparameters per backbone.

	Xception	EfficientNet-B4
Input size	299 × 299	380 × 380
Batch size	32	32
Head learning rate	1 × 10^−3^	5 × 10^−4^
Warm-up epochs	5	3
Unfrozen top-level children	6	4
Fine-tune LR	1 × 10^−5^	5 × 10^−6^
Max epochs	30	30

**Table 2:** Optuna search space for the EfficientNet-B4+SurgWound and EfficientNet-B4+RedScar+SurgWound configurations (100 trials per study, TPE sampler). Learning rates and weight decay are sampled on a logarithmic scale. Batch size is tuned within each unfreeze regime’s memory-safe range, because unfreezing B4’s MBConv body sharply raises activation memory and caps the batch that fits at 380^2^.

Hyperparameter	Type	Range/Choices
Head learning rate	log-float	10^−4^–3 × 10^−3^
Fine-tune (backbone) LR	log-float	10^−6^–10^−4^
Unfrozen top-level children	categorical	4, 5, 7
Warm-up epochs	integer	1–8
Weight decay	log-float	10^−6^–10^−3^
Batch size (unfreeze = 4)	categorical	8, 16, 32, 48
Batch size (unfreeze = 5 or 7)	categorical	8, 16

**Table 3:** Patient baseline characteristics.

**Baseline Characteristic**	**Mean ± SD**	***n* (%)**
Age (year)	57.4 ± 11.9	–
Sex (M/F)	–	55 (72)/21 (28)
LVEF (%)	54.1 ± 9.6	–
Euroscore (%)	1.7 ± 1.3	–
BMI (kg/m^2^)	28.0 ± 5.1	–
NYHA class (median)	II (IQR II–III)	–
**Type of Surgery**		
CABG	–	30 (39)
Valve	–	28 (37)
Aorta	–	11 (14)
Combined procedure	–	7 (9)
**Medical Characteristic**	**Mean ± SD**	***n* (%)**
Hypertension	–	62 (82)
Diabetes	–	21 (28)
COPD	–	11 (14)
Length of hospital stay (days)	9.7 ± 2.8	–
History of smoking	–	43 (57)
History of paroxysmal AF	–	14 (18)
Urgent surgery	–	8 (11)
Serum creatinine (mg/dL)	0.93 ± 0.19	–

Abbreviations: AF, atrial fibrillation; BMI, body mass index; CABG, coronary artery bypass grafting; COPD, chronic obstructive pulmonary disease; IQR, interquartile range; LVEF, left ventricular ejection fraction; NYHA, New York Heart Association; SD, standard deviation. Data are presented as *n* (%), mean ± SD, or median (IQR), as appropriate.

**Table 4:** Study adherence.

**Body Weight**	**Number of Records**	**Per Patient/Day**
1st week	477	0.90
2nd week	456	0.86
3rd week	435	0.82
4th week	326	0.61
**Arterial Blood Pressure + Heart Rate**	**Number of Records**	**Per Patient/Day**
1st week	942	1.77
2nd week	922	1.73
3rd week	826	1.55
4th week	721	1.36
**Surgical Wound Photos**	749	2.46 (per submitting patient/week)

Data are presented as number or number per patient per day or per week as appropriate. Per patient/day values for blood pressure and body weight are per enrolled patient (76 patients × 7 days) and are not adjusted for hospitalisation or death. Patients communicated with the study staff a total of 151 times with 69 phone calls and 82 text messages (SMS). No e-mail communication was used.

**Table 5:** Clinical events during the monitoring period (*n* = 76).

Event	*n* (%)
Completed the 30-day programme	75/76 (98.7)
Died (sudden death at home on day 15; cause not established)	1/76 (1.3)
Patients with at least one teleconsultation	76/76 (100)
Teleconsultations/Remote medical interventions	494/551
Referred to the outpatient clinic (next working day)	5/76 (6.6)
Surgical wound healing problems	4
Dyspnoea with pleural effusion	1
Referred to the emergency department	10/76 (13.2)
Discharged the same day (AF, sinus rhythm restored)	4
Admitted to hospital	6
Hospital admission	6/76 (7.9)
AF	3
Third-degree AV block, pacemaker implanted	2
High fever, suspected infective endocarditis (not confirmed)	1
Patients with AF	11/76 (14.5); 18 episodes
Wound complications (debridement, positive culture, antibiotics)	4/76 (5.3)
Attended the closing visit	75/75 (100)

Follow-up to day 30 or death was complete for all 76 patients. At the closing visit, all patients were in sinus rhythm, wound healing was normal, and echocardiography revealed no abnormalities. Abbreviations: AF, atrial fibrillation; AV, atrioventricular.

**Table 6:** Screening and tuning results. The twelve screened rows are 5-fold cross-validated validation means (used for selection; test set held out); the best value in each metric column (across the screened rows) is in bold. The final block is the Optuna-tuned EfficientNet-B4+RedScar+SurgWound model, shown as both its 5-fold CV validation performance and the held-out test performance of the ensemble of its five fold-specific models. Values in brackets for the held-out test set represent non-parametric 95% confidence intervals derived from *B* = 5000 stratified patient-clustered bootstrap resamples, accounting for intra-patient correlation across longitudinal image submissions. Inflamed is the positive class.

Training Data	AUPRC	ROC-AUC	Bal. Acc.	Recall	Prec.	F_1_	Acc.
**Xception—5-fold CV (validation)**							
study images only	0.497	0.806	0.721	0.587	0.381	0.419	0.825
+RedScar	0.605	0.835	0.700	0.680	0.254	0.351	0.722
+SurgWound	0.488	0.806	0.693	0.517	0.451	0.346	0.826
+RedScar+SurgWound	0.563	0.825	0.747	0.621	**0.527**	0.469	0.845
+RedScar-Inflamed	0.509	0.798	0.733	0.699	0.280	0.388	0.759
+RedScar-Infl.+SurgWound	0.532	0.811	0.732	0.560	0.406	0.454	**0.864**
**EfficientNet-B4—5-fold CV (validation)**							
study images only	0.608	0.826	0.737	0.755	0.264	0.379	0.725
+RedScar	0.568	0.855	0.755	0.805	0.269	0.394	0.716
+SurgWound	**0.652**	0.854	0.799	0.761	0.395	**0.509**	0.830
+RedScar+SurgWound	0.650	**0.888**	**0.801**	**0.846**	0.297	0.438	0.767
+RedScar-Inflamed	0.602	0.830	0.741	0.789	0.240	0.362	0.704
+RedScar-Infl.+SurgWound	0.621	0.856	0.766	0.741	0.329	0.442	0.788
**EfficientNet-B4+RedScar+SurgWound, Optuna-tuned (best-AUPRC)**							
validation (5-fold CV)	0.771	0.927	0.812	0.643	0.810	0.697	0.946
**test (held-out, 5-model ensemble)**	**0.747** **(0.402–1.000)**	**0.927** **(0.794–1.000)**	**0.852** **(0.643–1.000)**	**0.733** **(0.333–1.000)**	**0.733** **(0.429–1.000)**	**0.733** **(0.389–1.000)**	**0.946 (0.876–1.000)**

**Table 7:** Default vs. Optuna-found hyperparameters for EfficientNet-B4+RedScar+SurgWound (best-AUPRC trial). The decisive change is unfreezing the backbone (unfreeze: 4→7).

Hyperparameter	Default	Optuna (Best-AUPRC)
Head learning rate	5 × 10^−4^	2.4 × 10^−4^
Fine-tune (backbone) LR	5 × 10^−6^	5.9 × 10^−5^
Unfrozen children	4 (body frozen)	7 (entire backbone)
Warm-up epochs	3	4
Weight decay	0	2.4 × 10^−4^
Batch size	32	8

**Table 8:** Area-normalised relevance mass (ANRM) of the Grad-CAM++ explanations on the expert-annotated wound regions, for the 11 test images whose true class was Inflamed. ANRM = 1 is the uniform heatmap reference.

Image	ANRM
Leg 1-a	4.48
Leg 1-b	4.71
Leg 2-a	2.57
Leg 2-b	2.67
Leg 2-c	3.53
Leg 9-a	2.81
Leg 9-b	3.01
Leg 9-c	3.67
Leg 9-d	3.44
Sternotomy 9	1.59
Sternotomy 10	1.40
Mean	3.08
Median	3.01

Identifiers give the wound type and wound number; letters denote successive photographs of the same wound.

**Table 9:** Area-normalised relevance mass (ANRM) of Grad-CAM++ attribution maps for the four false-negative images, evaluated separately for the true (Inflamed) and predicted (Good) classes.

Image	Inflamed ANRM	Good ANRM
Sternotomy 5-a	1.58	1.50
Sternotomy 5-b	2.03	2.07
Sternotomy 5-c	2.25	1.85
Sternotomy 5-d	2.12	2.02
**Mean**	**2.00**	**1.86**

## Data Availability

The de-identified data supporting the findings of this study are available from the corresponding author on reasonable request. The wound image dataset is not publicly available owing to patient privacy constraints. The trained model and analysis code are available from the corresponding author on reasonable request.

## Supplementary Materials

The following supporting information can be downloaded at: https://www.mdpi.com/article/10.3390/jcm15218288/s1. File S1: Remote monitoring of patients after cardiac surgery—patient opinion (English translation of the 15-item patient acceptability questionnaire); File S2: TRIPOD+AI Checklist.

## Abbreviations

ABP	arterial blood pressure
AF	atrial fibrillation
AI	artificial intelligence
ANRM	area-normalised relevance mass
AUPRC	area under the precision–recall curve
BMI	body mass index
CABG	coronary artery bypass grafting
COPD	chronic obstructive pulmonary disease
CV	cross-validation
ECG	electrocardiogram
GPU	graphics processing unit
Grad-CAM	gradient-weighted class activation mapping
Grad-CAM++	generalised gradient-weighted class activation mapping
INR	international normalised ratio
IQR	interquartile range
LVEF	left ventricular ejection fraction
NYHA	New York Heart Association
RMA	relevance mass accuracy
RMI	remote medical intervention
ROC-AUC	area under the receiver operating characteristic curve
SD	standard deviation
TPE	tree-structured Parzen estimator
