# Supplementary material for: Artificial Intelligence-Enhanced Remote Monitoring After Cardiac Surgery: A Prospective Single-Centre Feasibility Study with Retrospective Wound Image Classification
Source: J Clin Med. 2026 Oct 24;15(21):8288. doi: 10.3390/jcm15218288 (PMC13763666; doi:10.3390/jcm15218288)
Supplement: Supplementary file 1 [file jcm-15-08288-s001.zip › File S1 Remote monitoring of patients after cardiac surgery—patient opinion.pdf]

Remote monitoring of patients after cardiac surgery – patient opinion

Please complete a short questionnaire and express your opinion about the study in which, for 30 days, we monitored your health parameters. The study is for scientific purposes only. Thank you for participating in the study and for your time.

According to your own feelings, please respond to the following statements on a scale from 1 to 7, where 1 – means strongly disagree, and 7 – strongly agree (place an x in each row).

Item	1 – strongly disagree	2	3	4	5	6	7 – strongly agree
1. I believe that using the devices provided is simple							
2. I believe that it is easy to learn how to use these devices							
3. Overall, I believe that using remote monitoring of health status is convenient							
4. This solution may increase my level of convenience in accessing medical care							
5. Overall, I believe that the solution is very useful							
6. As the first among people in my surroundings, I try new technologies							
7. I like trying to use new technologies							
8. I am open to new ideas							
9. My family encourages and supports me in using such a solution							
10. My friends encourage and support me in using such a solution							

11. Medical staff encourage and support me in using such a solution							
12. The media promote the use of this type of solution							
13. In my health situation, I would be willing to use such a solution in the future							
14. While using the solution, I felt safer							
15. People close to me take an interest in my health status on a daily basis							
