# Supplementary material for: Artificial Intelligence-Enhanced Remote Monitoring After Cardiac Surgery: A Prospective Single-Centre Feasibility Study with Retrospective Wound Image Classification
Source: J Clin Med. 2026 Oct 24;15(21):8288. doi: 10.3390/jcm15218288 (PMC13763666; doi:10.3390/jcm15218288)
Supplement: Supplementary file 1 [file jcm-15-08288-s001.zip › File_S2_TRIPOD-AI_checklist.pdf]

File S2. TRIPOD+AI Checklist

Manuscript: Artificial Intelligence-Enhanced Remote Monitoring After Cardiac Surgery: A Prospective Single-Centre Feasibility Study with Retrospective Wound-Image Classification

Corresponding author: Jarosław Bis, MD — Department of Cardiac Surgery, Medical University of Silesia, Katowice, Poland

Reporting guideline: Collins GS, Moons KGM, Dhiman P, et al. TRIPOD+AI statement: updated guidance for reporting clinical prediction models that use regression or machine learning methods. BMJ 2024;385:e078378.

Note on scope. The prediction model reported in this manuscript is a binary image classifier whose target is the clinicians' visual inflamed/not-inflamed judgment of a wound photograph. It was developed and evaluated retrospectively as a proof of concept and did not inform clinical care during the monitoring programme. Items concerning prospective deployment, health-economic evaluation and external validation are therefore reported as not applicable or not done, with reasons.

Locations are given as manuscript sections (subsection headings in quotation marks), so that they remain valid if pagination changes during production.

Item	Checklist item	Location in manuscript	Where / how addressed
**Title**			
1	Identify the study as developing or evaluating a multivariable prediction model, the target population, and the outcome to be predicted.	Title	Title identifies the AI-enhanced remote monitoring programme, the population (after cardiac surgery), the prospective single-centre feasibility design and the retrospective wound-image classification.
**Abstract**			
2	Provide a structured summary of objectives, methods, results and conclusions.	Abstract	Structured abstract (Background/Objectives, Methods, Results, Conclusions), including the principal test-set metrics with 95% confidence intervals.
**Introduction**			
3a	Explain the medical context and rationale for developing or evaluating the model.	Introduction	Readmission burden after cardiac surgery and the rationale for remote monitoring with automated image triage.
3b	Describe the target population and the intended use of the model in context.	Introduction; Conclusions	Intended use stated as a human-in-the-loop triage layer prioritising wound photographs for clinician review.
4	Specify the objectives, including whether the study describes model development, validation, or both.	Introduction (aims); Methods, "Investigational AI-based wound-image classifier"	Development and internal evaluation on a patient-disjoint held-out test set; validation on a larger, multicentre dataset stated as the next step.
**Methods — Data**			
5a	Describe the sources of data separately for development and evaluation.	Methods, "Task and data"	Study photographs (735 images of 88 wounds from 71 patients) collected prospectively during the monitoring programme; optional enrichment with two public datasets (SurgWound, RedScar) appended to the training part of each fold only, never to validation folds or the test set.
5b	Specify the dates of data collection.	Methods, "Study design"	Recruitment March 2024 to November 2025; photographs collected during each patient's 30-day monitoring period.
5c	Specify whether the study was linked to a registry or trial.	Methods, "Study design"	Not applicable — single-arm feasibility study, not a registered clinical trial.
6a	Describe eligibility criteria for participants.	Methods, "Study design"; "Recruitment and feasibility outcomes"	Camera-equipped smartphone, self-reported ability to operate the devices, discharge home 7–10 days after surgery, agreement to teleconsultations; exclusion for psychoactive substance addiction.
6b	Report details of any treatments received.	Methods, "Study procedure"; Results, "Teleconsultations and clinical events"	Standard postoperative care; teleconsultation-driven interventions reported (494 teleconsultations, 551 remote medical interventions).
**Methods — Outcome**			
7a	Clearly define the outcome that is	Methods, "Task and data"	Binary Inflamed vs Good label: the clinicians'

Item	Checklist item	Location in manuscript	Where / how addressed
	predicted, including how and when assessed.		visual judgment of each photograph, graded on the Southampton wound assessment scale (grade IId or higher labelled Inflamed).
7b	Report any actions to blind assessment of the outcome.	Methods, “Task and data”	Two specialist cardiac surgeons graded each photograph independently, blinded to each other’s assessment and to the wound’s clinical outcome; all labelling preceded model development.
7c	If applicable, describe the reference standard and its reliability.	Methods, “Task and data”; Results, “Investigational wound-image classifier”	Consensus of two independent readers, with adjudication of the 16 discordant images (2.2%) by a third specialist cardiac surgeon. Agreement: 97.8% (719/735), Cohen’s κ 0.89 (95% CI 0.83–0.94), positive specific agreement 89.9%, Gwet’s AC1 0.97. Reference standard external to the image for the confirmed-complication analysis: surgical debridement, positive bacterial culture and antibiotic treatment.
**Methods — Predictors**			
8a	Describe the predictors/input data, including how and when measured.	Methods, “Monitored health parameters / Devices”; “Task and data”	Wound photographs taken and submitted by the patients themselves with their own smartphones on alternate days.
8b	Describe any data pre-processing and harmonisation.	Methods, “Data augmentation”	All images: EXIF orientation correction, aspect-preserving resizing with letterboxing, Shades-of-Gray white balance ($p = 6$) and ImageNet normalisation. Augmentation (random resized crop, horizontal flip, rotation ±10°, translation ±5%, colour jitter) applied to training images only.
**Methods — Sample size / Missing data**			
9	Explain how the study size was arrived at.	Methods, “Task and data”; Discussion, “Limitations”	No formal sample-size calculation. The image set was determined by the photographs collected during the feasibility programme (convenience sample); the small number of positive cases is stated as a limitation.
10	Describe how missing data were handled.	Methods, “Task and data”; Results, Figure 1	No imputation. Of 749 photographs received, 735 were analysed; 14 photographs from 6 patients were excluded (9 taken after surgical debridement, 5 of insufficient quality), and no patient or wound was excluded entirely. Five enrolled patients submitted no photographs.
**Methods — Analytical methods**			
11a	Describe how the data were used (training, tuning, evaluation) and how splits were made.	Methods, “Task and data”; “Checkpoint selection and evaluation”; “Screening, selection and tuning procedure”	Patient-level split: held-out test set of 147 images from 15 patients, separated first and never used in development; development on the remaining 588 images from 56 patients by patient-disjoint 5-fold cross-validation, used for screening, hyperparameter optimisation and validation.
11b	Specify the model type and modelling approach.	Methods, “Backbones and training”; “Screening, selection and tuning procedure”; “Statistical analysis”	ImageNet-pretrained convolutional backbones (Xception, EfficientNet-B4) with a linear classification head; two-phase training. Final model: ensemble of the five fold models of the tuned EfficientNet-B4+RedScar+SurgWound configuration, with averaged predicted probabilities thresholded at 0.5. Software: Python 3.15.0, PyTorch 2.14.1 (timm library), scikit-learn 1.9.1 and Optuna; statistical analyses in R 4.6.1.
11c	Describe how hyperparameters were tuned.	Methods, “Hyperparameter optimisation with	Optuna with the Tree-structured Parzen Estimator sampler, 100 trials for each of the two leading configurations; objective = mean

Item	Checklist item	Location in manuscript	Where / how addressed
		Optuna"; "Screening, selection and tuning procedure"; Table 2	5-fold cross-validated validation AUPRC; test set never used in the search. The final configuration was chosen on validation data only, before the test set was evaluated.
11d	Describe how class imbalance was addressed.	Methods, "Handling class imbalance"	Inverse-frequency class weighting of the cross-entropy loss (0.55–0.56 Good, 4.54–5.07 Inflamed, depending on the fold); AUPRC used for checkpoint selection.
11e	Specify all measures used to assess model performance.	Methods, "Checkpoint selection and evaluation"; "Statistical analysis of results on test set"	AUPRC (computed as average precision; primary) and ROC-AUC; balanced accuracy, recall, precision, F_1 and overall accuracy at the default threshold; wound-level sensitivity. 95% confidence intervals from 5000 stratified patient-level cluster bootstrap resamples.
11f	Describe any model updating or recalibration.	—	Not applicable — no updating or recalibration was performed.
12	Describe any approaches to evaluate fairness or performance in subgroups.	Discussion, "Limitations"	Not done. Subgroup analyses (for example by sex or wound type) were not performed because of the small number of positive cases; the absence of a sex- or gender-stratified analysis is stated in the Limitations.
13	Describe approaches to model explainability or interpretability.	Methods, "Explainability analysis"	Grad-CAM++ attribution maps of the five-model ensemble compared with rectangular expert masks enclosing the wound, drawn by a single cardiac surgeon before the maps were viewed; spatial agreement scored by area-normalised relevance mass (ANRM), where ANRM > 1 indicates concentration inside the expert region.
**Results**			
14a	Describe the flow of participants/data, including numbers included and excluded.	Results, "Participants and feasibility"; Figure 1	85 screened, 9 ineligible, 76 enrolled, 75 completed, 1 died; 749 photographs received from 71 patients, 735 analysed (88 wounds).
14b	Report the characteristics of the participants.	Results, "Participants and feasibility"; Table 3	Table 3 (age, sex, LVEF, EuroSCORE, BMI, NYHA class, procedure type, comorbidities).
15	Report the number of outcome events.	Methods, "Task and data"; Results, "Investigational wound-image classifier"	78 Inflamed of 735 images (10.6%), from 28 wounds of 28 patients; 15 Inflamed images in the test set, from 6 wounds of 6 patients; 4 confirmed wound complications in the cohort, 1 of them in the test set.
16	Report model performance with confidence intervals where appropriate.	Results, "Investigational wound-image classifier"; Table 6; Figures 7–8	Test set: AUPRC 0.747 (95% CI 0.402–1.000), ROC-AUC 0.927 (0.794–1.000), balanced accuracy 0.852 (0.643–1.000), recall 0.733 (11/15; 0.333–1.000), precision 0.733 (0.429–1.000), F_1 0.733 (0.389–1.000), accuracy 0.946 (0.876–1.000). Wound-level sensitivity 5/6. The one confirmed wound complication in the test set was flagged.
17	Report the results of any fairness or subgroup evaluation.	—	Not done — see item 12.
18	Report results of explainability analyses.	Results, "Explainability"; Tables 8–9; Figure 9	ANRM exceeded 1 in all 11 correctly classified Inflamed test images (range 1.40–4.71; mean 3.08, median 3.01) and in the four false-negative images (mean 2.00 for the Inflamed and 1.86 for the Good class).
**Discussion**			
19	Discuss limitations, including sources of potential bias and non-generalisability.	Discussion, "Limitations"	Single-centre, single-arm design; small number of positive test cases (15 images from 6 patients) and wide confidence intervals; single-centre images labelled by the same readers; label noise and domain shift from the external training data; image-derived primary reference standard; partial entanglement of

Item	Checklist item	Location in manuscript	Where / how addressed
			complication ascertainment with image appearance; only one confirmed complication in the test set; retrospective application; explainability shows alignment, not causal faithfulness.
20	Give an overall interpretation of the results, considering objectives and prior evidence.	Discussion, “Main Findings”; “Comparison with Previous Studies”	Results framed as a hierarchy of three claims of decreasing evidential strength; compared with the WISDOM AI study and the Mayo Clinic multicentre study.
21	Discuss the potential clinical use of the model and implications for future research.	Discussion; Conclusions	Human-in-the-loop triage prioritising images for clinician review; validation on a larger, multicentre dataset and multicentre randomised studies identified as next steps.
**Other information**			
22	Provide information about the availability of data, code and the model.	Data Availability	De-identified data, trained model and analysis code available from the corresponding author on reasonable request; wound images not publicly available owing to patient privacy.
23	Provide details of funding and the role of the funders.	Funding	Statutory funds of the Medical University of Silesia (BNW-1-121/N/4/K); the funder had no role in design, analysis, interpretation, writing or the decision to publish.
24	Report conflicts of interest.	Conflicts of Interest	The authors declare no conflicts of interest.
25	Provide details of ethical approval and participant consent.	Methods, “Ethics approval and consent to participate”; Institutional Review Board Statement	Bioethics Committee of the Medical University of Silesia (BNW/NWN/0052/KB1/9/I/24, 19 March 2024). Written informed consent covering participation and the retrospective use of wound photographs for algorithm development and publication.

Completed by the corresponding author.
